# Polyhydroxy cucurbitane triterpenes from *Hemsleya penxianensis* tubers

**DOI:** 10.1038/s41598-019-48365-0

**Published:** 2019-08-14

**Authors:** Zhaocui Sun, Meigeng Hu, Nailiang Zhu, Xiaowei Huo, Xiaolei Zhou, Zhonghao Sun, Junshan Yang, Guoxu Ma, Xudong Xu

**Affiliations:** 1 Key Laboratory of Bioactive Substances and Resource Utilization of Chinese Herbal Medicine, Ministry of Education, Institute of Medicinal Plant Development, Peking Union Medical College and Chinese Academy of Medical Sciences, Beijing, 100193 People’s Republic of China; 2 College of Pharmaceutical Science, Key Laboratory of Pharmaceutical Quality Control of Hebei Province, Hebei University, Baoding, 071002 China

**Keywords:** Secondary metabolism, Environmental chemistry, Structure elucidation, Solution-state NMR, Medicinal chemistry

## Abstract

Ten new cucurbitane triterpenoids, hemsleyacins A–J (**1**–**10**), together with three known cucurbitane triterpenoids, dihydrocucurbitacin F (**11**), scandenogenin D (**12**), and jinfushanencin F (**13**), were separated from ethanolic tuber extracts of *Hemsleya penxianensis*. The absolute configurations of the new compounds were established based on NMR, HRESIMS, and CD spectra. Compounds **7** and **10–12** were evaluated in terms of their antifeedant activity against *Plutella xylostella* larvae. The result showed that compound **10** exhibited potent antifeedant activity against *P*. *xylostella* larvae after 48 h of treatment. Furthermore, the MTT test showed that compound **11** exhibited potent inhibition toward the UMUC-3 and T24 cell lines with IC_50_ values of 29.12 and 35.62 μM, respectively, compared to the positive control cisplatin IC_50_ values of 8.27 and 13.72 μM. Western blot analysis revealed that compound **11** treatments substantially inhibited the phosphorylation of IκBα.

## Introduction

Different plant species contain unique secondary compounds that act as chemical defense against insects and other pathogens. Although plants can resist pest invasion through their own defense mechanisms, with the changes in the ecological environment changes, the role of plant protection agents has become increasingly important. Recent studies have reported significant antifeedant effects of cucurbitane-type compounds on insects and the inhibition of cancer cells^1–4^. In our previous studies, we isolated various cucurbitane-type compounds from *Hemsleya penxianensis* that exhibited significant inhibitory activity against cancer cell lines^4,5^.

**Figure 1:**
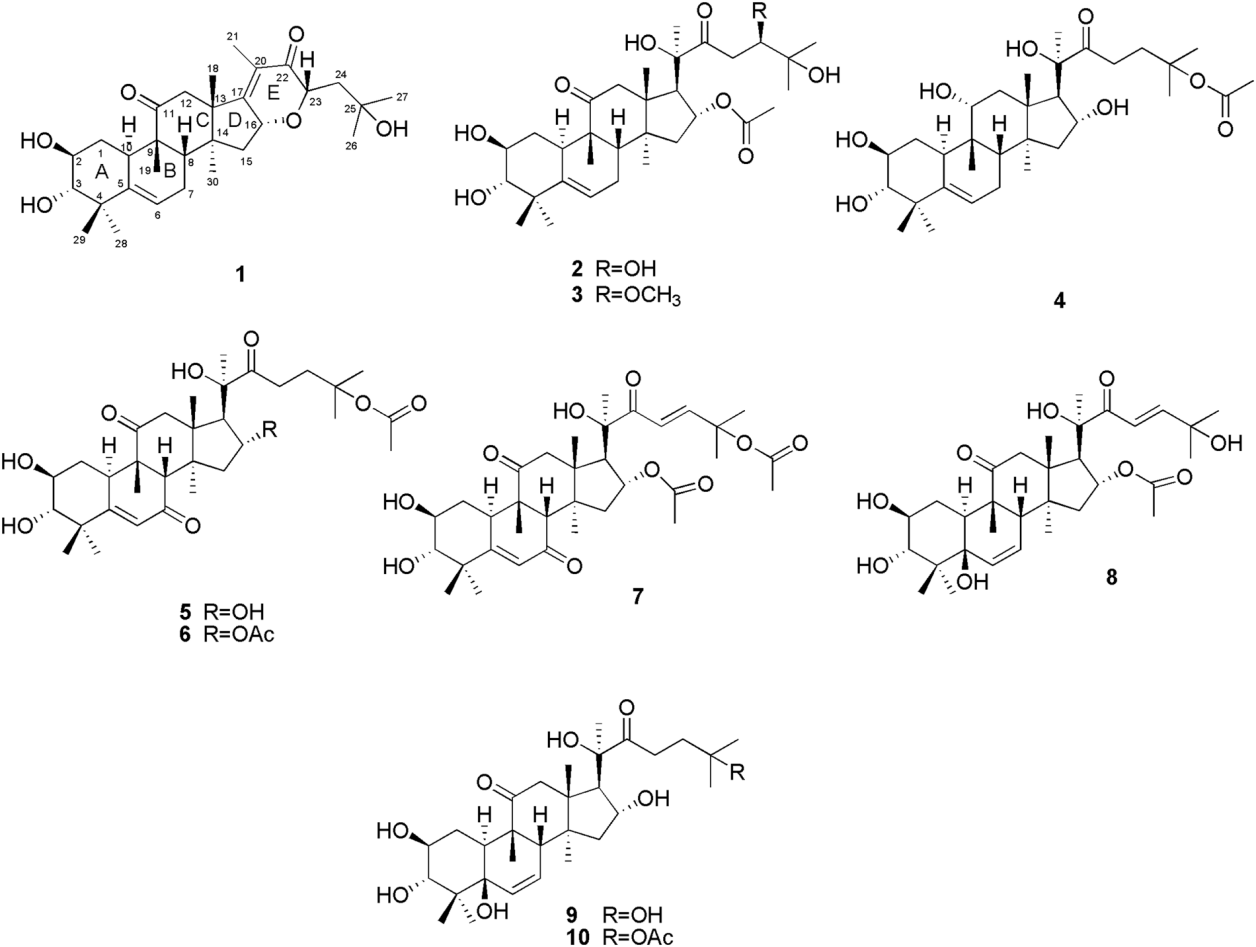
Structures of compounds **1**–**10**.

*Hemsleya penxianensis*, called “Xuedan” in Chinese, is a member of the Cucurbitaceae family that is found in the Sichuan, Yunnan, and Guizhou provinces in China. To discover potential antifeeding products, we evaluated a 95% ethanol extract of *H*. *penxianensis*, obtaining 10 new cucurbitane-type triterpene hemsleyacins A–J (**1**–**10**) and three known cucurbitane-type triterpenes, namely, dihydrocucurbitacin F^6^ (**11**), scandenogenin D^7^ (**12**), and jinfushanencin F^8^ (**13**) (Fig. 1). Their structures were elucidated based on an examination of their spectra using 600 MHz nuclear magnetic resonance (NMR) spectroscopy [^1^H and ^13^C-NMR, homonuclear correlation spectroscopy (COSY), heteronuclear single quantum coherence spectroscopy (HSQC), heteronuclear multiple bond correlation (HMBC), and nuclear overhauser spectroscopy (NOESY)], infrared radiation (IR), ultraviolet spectroscopy, mass spectrometry (MS), and circular dichroism (CD). Compounds **7** and **10**–**12** were tested for antifeedant activity against *Plutella xylostella* larvae. The results showed that these compounds exhibited potent antifeedant activity against *P*. *xylostella* larvae after 48 h of treatment. Furthermore, compounds **1**–**13** were assessed *in vitro* against two bladder cancer cell lines, UMUC-3 and T24.

## Results and Discussion

Different plant species contain unique secondary compounds that act as chemical defense against insects and other pathogens. Although plants can resist pest invasion through their own defense mechanisms, with the changes in the ecological environment changes, the role of plant protection agents has become increasingly important. Recent studies have reported significant antifeedant effects of cucurbitane-type compounds on insects and the inhibition of cancer cells^1–4^. In our previous studies, we isolated various cucurbitane-type compounds from *Hemsleya penxianensis* that exhibited significant inhibitory activity against cancer cell lines^4,5^.

*Hemsleya penxianensis*, called “Xuedan” in Chinese, is a member of the Cucurbitaceae family that is found in the Sichuan, Yunnan, and Guizhou provinces in China. To discover potential antifeeding products, we evaluated a 95% ethanol extract of *H*. *penxianensis*, obtaining 10 new cucurbitane-type triterpene hemsleyacins A–J (**1**–**10**) and three known cucurbitane-type triterpenes, namely, dihydrocucurbitacin F^6^ (**11**), scandenogenin D^7^ (**12**), and jinfushanencin F^8^ (**13**) (Fig. 1). Their structures were elucidated based on an examination of their spectra using 600 MHz nuclear magnetic resonance (NMR) spectroscopy [^1^H and ^13^C-NMR, homonuclear correlation spectroscopy (COSY), heteronuclear single quantum coherence spectroscopy (HSQC), heteronuclear multiple bond correlation (HMBC), and nuclear overhauser spectroscopy (NOESY)], infrared radiation (IR), ultraviolet spectroscopy, mass spectrometry (MS), and circular dichroism (CD). Compounds **7** and **10**–**12** were tested for antifeedant activity against *Plutella xylostella* larvae. The results showed that these compounds exhibited potent antifeedant activity against *P*. *xylostella* larvae after 48 h of treatment. Furthermore, compounds **1**–**13** were assessed *in vitro* against two bladder cancer cell lines, UMUC-3 and T24.

## Results and Discussion

### Isolation and structural elucidation

The air-dried tubers of *H*. *penxianensis* were extracted three times with EtOH. The resulting extract was subjected to column chromatography and purified by semipreparative high-performance liquid chromatography (HPLC), affording 10 new and three known polyhydroxy cucurbitane-type triterpenes (**1**–**13**).

Compound **1** was obtained as a white amorphous powder. Its IR spectrum had the absorption signals of hydroxy groups at 3396 cm^−1^ and carbonyl groups at 1662 cm^−1^. A molecular formula of C_30_H_44_O_6_ was deduced based on the [M + Na]^+^ ion peak at *m/z* 523.3333 (calculated for C_30_H_44_O_6_Na, 523.3036) in positive HR-ESIMS, necessitating 9 degrees of unsaturation. The ^1^H-NMR spectrum revealed the existence of eight tertiary methyl groups at *δ*_H_ 0.84, 1.09, 1.10, 1.16, 1.28, 1.44, 1.73, and 2.36 (each s), showing correlations with C-18 (*δ*_C_ 21.4), C-27 (*δ*_C_ 28.5), C-26 (*δ*_C_ 28.4), C-29 (*δ*_C_ 22.6), C-19 (*δ*_C_ 21.0), C-30 (*δ*_C_ 25.9), C-28 (*δ*_C_ 20.2), and C-21 (*δ*_C_ 15.0), respectively, in the HSQC spectrum. In addition, four oxygenated methines at *δ*_H_ 3.89 (1 H,d, *J* = 9.0 Hz, H-2), 3.37 (1 H,d, *J* = 9.0 Hz, H-3), 3.93 (1 H, m, H-16), and 5.11 (1 H, t, *J* = 6.0 Hz, H-23), and an olefinic proton signal at *δ*_H_ 5.74 (1 H, d, *J* = 6.0 Hz, H-6), were observed. The ^13^C NMR and ^13^C APT spectra exhibited 30 carbon resonances in total, corresponding to eight methyls, five methylenes, and seven methines, including two oxymethines at *δ*_C_ 56.8 (C-16) and *δ*_C_ 71.7 (C-23), and carbons at *δ*_C_ 119.0 (C-6), *δ*_C_ 44.4 (C-8),*δ*_C_ 34.7 (C-10), *δ*_C_ 56.8 (C-16), and *δ*_C_ 71.7 (C-23), and 10 quaternary carbons. Taken together, these data were indicative of a cucurbitacin triterpene^4,9–11^.

**Figure 2:**
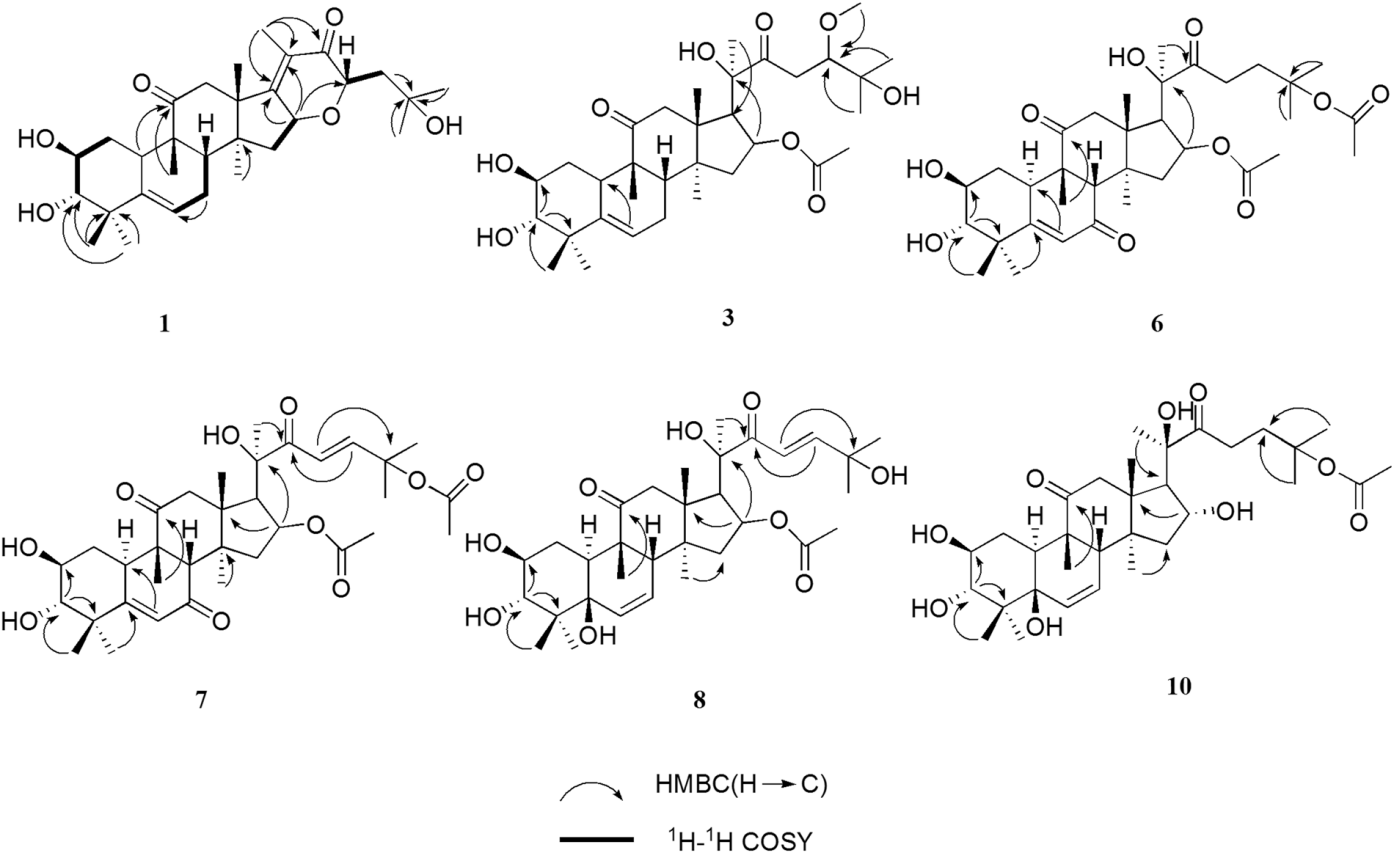
Key HMBC and ^1^H- ^1^H correlations for compounds **1**, **3**, **6–8**, and **10**.

**Figure 3:**
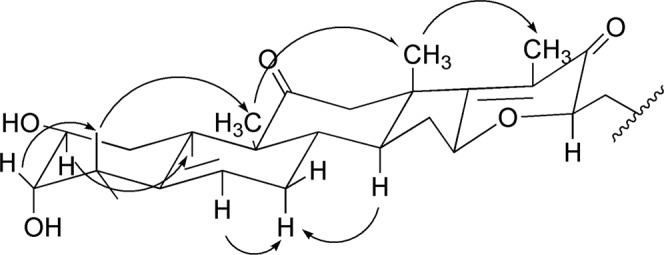
Key NOESY correlations of compound **1**.

**Figure 4:**
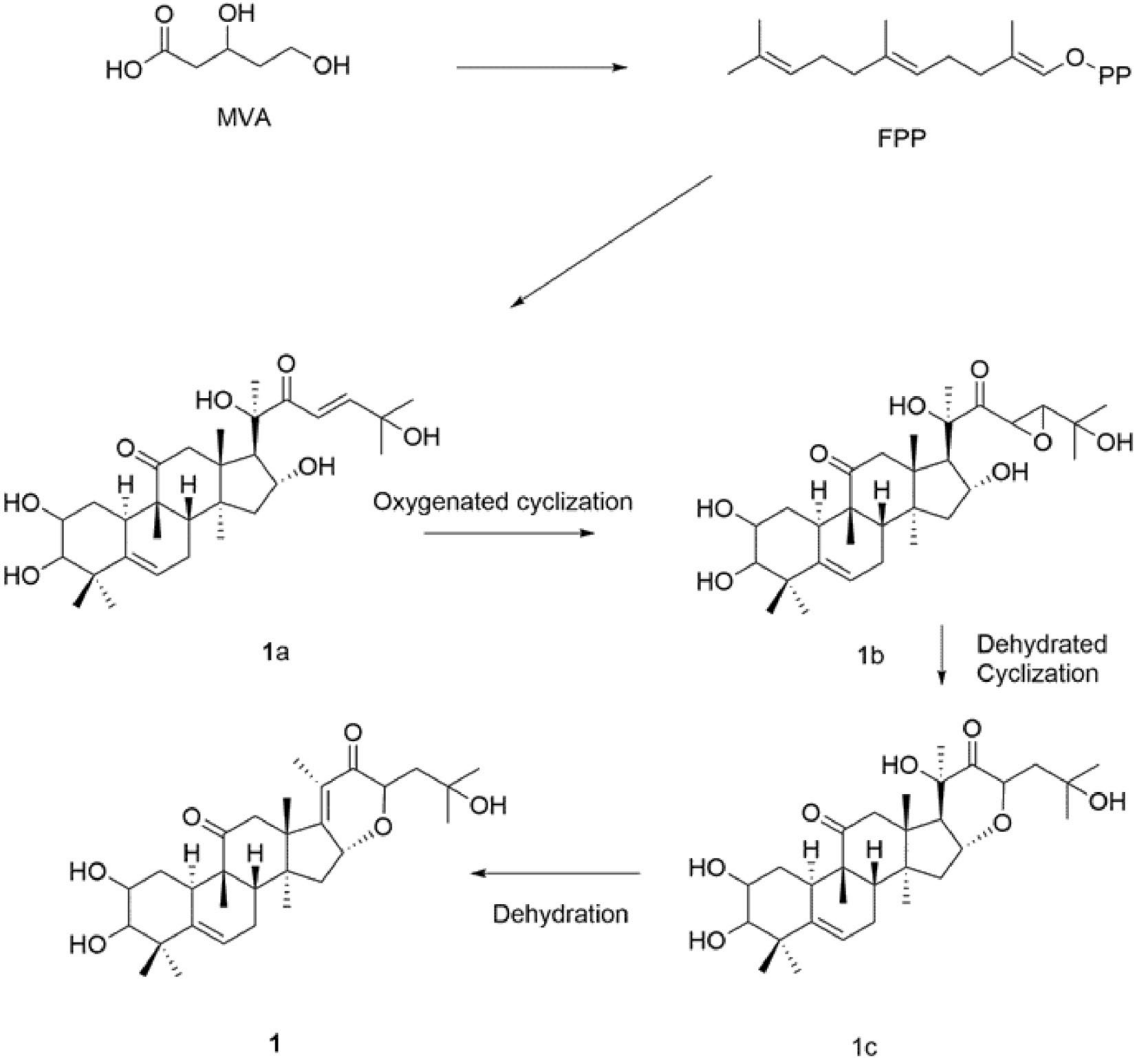
Hypothetical biosynthetic pathways for compound 1.

The proposed structure was further confirmed by an evaluation of the 2D NMR data, including ^1^H-^1^H COSY, HSQC, and HMBC experiments. In the HSQC spectra, the olefinic proton signal at H-6 (*δ*_H_ 5.71) was correlated with the olefinic carbon signal at C-6 (*δ*_C_ 119.0). Together with the HMBC correlations **(**Fig. 2**)** from H-7 (*δ*_H_ 1.87) and H-8 (*δ*_H_ 1.93) to the olefinic carbon (C-6, *δ*_C_ 119.0), this suggested that the double bond was linked to C-5 and C-6. One of the carbonyl carbon signals at C-11 (*δ*_C_ 213.7) was verified by the HMBC correlations from both H_3_−19 and H-10, while the other carbonyl carbon signal at C-22 was correlated with H_3_-21. The positions of the methyl groups were established based on the HMBC correlations from *δ*_H_ 1.16 (CH_3_-29) and *δ*_H_ 1.73 (CH_3_-28) to C-3 (*δ*_C_ 81.9) and C-4 (*δ*_C_ 43.2), *δ*_H_ 1.28 (CH_3_-19) to C-11(*δ*_C_ 213.7), *δ*_H_ 1.44 (CH_3_-30) to C-14 (*δ*_C_ 54.3), *δ*_H_ 2.36 (CH_3_-21) to C-17 (*δ*_C_ 121.8), C-20 (*δ*_C_ 146.6), and C-22 (*δ*_C_ 200.4), and *δ*_H_ 1.10 (CH_3_-26) and *δ*_H_ 1.09 (CH_3_-27) to C-25 (*δ*_C_ 79.0). An examination of the HMBC spectrum revealed that the cross-peaks from CH_3_-21 to C-17 (*δ*_C_ 121.8), C-20 (*δ*_C_ 146.6), and C-22 (*δ*_C_ 200.4) were indicative of an *α*,*β*-unsaturated carbonyl moiety. Furthermore, the correlations from H-16 (*δ*_H_ 3.93) to C-17 (*δ*_C_ 121.8), and C-20 (*δ*_C_ 146.6) and C-23 (*δ*_C_ 71.7) suggested that the six-membered E-ring was closed via C-16/O/C-23, which is consistent with the degree of unsaturation. Finally, three hydroxyl groups were present at C-2, C-3, and C-25, respectively, based on the downfield chemical shifts of *δ*_C_ 71.2, 81.9, and 79.0 together with the molecular formula C_30_H_44_O_6_ above. The NOESY experiment and coupling constants determined the relative configuration of compound **1** in which NOESY correlations of H-19 and H-3, and H-10 and H-2 suggested an α-configuration for OH-3 and *β*-configuration for OH-2 **(**Fig. 3**)**. The ^3^*J* coupling constant (*J* = 9.0 Hz) also verified the antiperiplanar conformation between H-2 and H-3. Moreover, the observed NOESY cross-peaks of H-16/H-23 and H-16/Me-18 indicated the *β*-orientation of H-16 and H-23. The identical CD spectra of compound **1** and cucurbitacin IIa (300 nm, Δε4.1; 200.0 nm, Δε18.6) whose absolute configuration has been fully determined, indicated the *S*, *R*, *R*, *R*, and *S* configurations of C-8, C-9, C-10, C-13, and C-14, respectively. Compound **1** has a new cucurbitacin triterpene skeleton with an *α*, *β*-unsaturated carbonyl six-membered ring through an oxygen bridge between C-16 and C-23. Biogenetically, compound **1** could be converted from the hypothetical precursor **1a** (Fig. 4). Oxidation of the C-23/C-24 double bond will produce the epoxy derivative **1b**. Dehydration in C-16 forms a six-membered ring (E-ring) in **1c**. Then, **1c** immediately undergoes dehydration and finally generates compound **1**. Thus, the structure of **1** was identified and it was called hemsleyacin A.

Compound **2** was isolated as a white powder and had the molecular formula C_32_H_50_O_9_ based on HR-ESIMS (*m/z* 601.3369, calculated for C_32_H_50_O_9_Na [M + Na]^+^, 601.3353). The ^1^H NMR signals indicated the appearance of nine methyl groups at *δ*_H_ 1.18 (3 H, s), 1.20 (3 H, s), 1.65 (3 H, s), 1.56 (3 H, s), 1.54 (3 H, s), 1.28 (3 H, s), 1.35 (3 H, s), 1.51 (3 H, s), and 2.17 (3 H, s); four oxygenated methines at *δ*_H_ 4.15 (1 H, m), 3.44 (1 H, d, *J* = 9.0 Hz), 5.84 (1 H, d, *J* = 7.8 Hz), and 4.60 (1 H, d, *J* = 9.6 Hz); and an olefinic proton at *δ*_H_ 5.73 (1 H, d, *J* = 6.0 Hz). The ^13^C NMR spectrum contained 32 signals, including nine angular methyls, five methylenes, seven methines (one *sp*^2^ methines and four oxygenated methines), and 11 quaternary carbons (four *sp*^3^ carbons, one *sp*^2^ carbon, four carbonyl carbons, and two oxygenated carbons). **1** Based on a comparison of their NMR data, compound **2** has the same tetracyclic triterpenic nucleus as, but they differ in their side chains. The NMR spectral data indicated that the ring of the side chain in compound **1** was cracked in compound **2**, and an acetyl group was present at C-16 as was confirmed by the HMBC correlations from H-16 (*δ*_H_ 5.84) to an oxygenated carbon *δ*_C_ 171.0. Furthermore, the data suggested that the double bond at C-17/C-20 had disappeared, and hydroxyl groups were located at C-20 and C-24, respectively. Further analysis of the HMBC data, the correlations from H-26 and H-27 to *δ*_C_ 75.0 (C-24), H-21 to *δ*_C_ 55.2 (C-17) confirmed the above deduction. The cross peaks of H-17/Me-21 and H-16/Me-30/H-20 in the NOESY spectrum suggested that the side chain H-16, and H-20 were *β*-oriented, respectively. Compared with the carbon data of leucopaxillones A at C-24 (*δ*_C_ 75.0), the relative configuration of H-24 was determined as that of *α*-orientation^12^. Thus, the structure of compound **2** was determined and it was called hemsleyacin B.

Compound **3** was separated as a white amorphous powder and had a molecular formula of C_33_H_52_O_9_ based on HR-ESIMS (*m/z* 615.3527 [M + Na]^+^, calculated for C_33_H_52_O_9_Na, 615.3509). The ^1^H NMR and ^13^C APT data were similar to those of **2** (Tables 1 and 2). One difference was that the carbon signal of **2** at C-24 (*δ*_C_ 75.0) was moved downfield by *δ*_C_ 85.0 (C-24) in **3**, and the molecular weight of **3** in HR-ESIMS was larger by 14 units than that of **2**, both of which suggested that the hydroxyl group at C-24 in **2** was replaced by an oxymethyl group in **3**. The NMR spectra revealed an extra proton signal at *δ*_H_ 3.62 (3 H, s) and a carbon signal at *δ*_C_ 60.5, and the HMBC spectrum (Fig. 2) revealed a correlation between *δ*_H_ 3.62 (3 H, s) and *δ*_C_ 85.0 (C-24), supporting the deduction above. The relative configuration of **3** was consistent with **2** based on a comparison of their NOESY spectral data. Therefore, the structure of compound **3** was determined, and it was called hemsleyacin C.

**Table 1:** ^1^H NMR Spectroscopic Data (600 MHz, in Pyridine-*d*_5_) for Compounds **1**–**10** (*δ*_H_ in ppm, *J* in Hz).

No.	1	2	3	4	5	6	7	8	9	10
1	2.40, m	2.42, m	2.44, m	3.08, m	2.60, m	1.64, m	2.65, m	2.63, m	2.62, m	2.62, m
	2.16, m	1.54, m	1.45, m	3.36, m	1.68, m	2.21, m	1.67, m	2.15, m	2.26, m	2.23, m
2	3.89, d (9.0)	4.15, m	4.12, m	4.29, m	4.16, m	4.15, m	4.14, m	4.22, d (12.0)	4.22, m	4.19, m
3	3.37, d (9.0)	3.44, d (9.0)	3.42, br d (9.0)	3.47, d (9.0)	3.53, d (8.4)	3.54, d (9.0)	3.54, d (9.0)	4.32, d (9.0)	4.37, m	4.37, d (9.0)
6	5.71, d (6.0)	5.73, d (6.0)	5.73, d (6.0)	5.70, d (6.0)	6.63, s	6.47, s	6.46, s	6.37, d (9.0)	6.31, d (10.4)	6.32, d (9.0)
7	1.87, m; 2.58, m	2.28, m; 1.78, m	1.83, m; 2.31, m	2.40, m; 2.48, m				5.65, m	5.74, m	5.74, m
8	1.93, m	1.87, m	1.88, m	1.82, m	2.80, s	2.75, s	2.74, s	2.35, m	2.37, m	2.38, m
10	2.45, m	2.66, m	2.69, m	3.08, m	3.26, m	3.23, m	3.24, m	2.54, m	2.62, m	2.45, m
11				4.47, m						
12	3.65, d (12.0)	2.76, d (15.0)	2.81, d (15.0)	2.33, d (15.0)	3.11 d (15.0)	2.90 d (15.0)	2.91, d (15.0)	2.95, m	3.00, d (9.0)	3.05, d (15.0)
	2.89, d (12.0)	3.16, d (15.0)	3.15, d (15.0)	2.48, d (15.0)	2.93 d (15.0)	3.33 d (15.0)	3.30, d (15.0)	2.92, m	2.90, d (9.0)	2.89, m
15	1.90, m	1.52, m	1.53, d (12.0)	1.67 d (12.0)	2.62, m	1.68, m	1.68, m	2.23, m	2.07, m	2.08, m
	2.11, m	2.03, m	2.06, d (12.0)	1.88 d (12.0)	1.85, m	2.77, m	2.77, m	1.67, m	1.81, m	1.82, d (12.6)
16	3.93, m	5.84, t (7.8)	6.03, m	4.97, m	4.91, m	6.07, t (7.8)	6.02, t (7.8)	5.88, t (12.0)	4.95, m	4.95, m
17		3.04 d (7.8)	3.15, d (7.8)	2.92, d (5.4)	2.91, d (7.8)	3.04 d (7.8)	2.99, d (7.8)	2.83, m	2.92, m	2.87, m
18	0.84, s	1.18, s	1.14, s	1.39, s	1.25, s	1.19, s	1.22, s	1.19, s	1.22, s	1.23, s
19	1.28, s	1.20, s	1.20, s	1.37, s	1.27, s	1.25, s	1.24, s	1.61, s	1.64, s	1.64, s
21	2.36, s	1.65, s	1.62, s	1.68, s	1.60, s	1.56, s	1.58, s	1.55, s	1.60, s	1.64, s
23	5.10, t (7.2)	3.41 d (8.4)	3.36, d (8.4)	1.68, m	3.28, m	3.03, m	7.26, d (9.0)	7.45, m	3.52, m	3.35, m
		3.57 d (8.4)	3.48, d (8.4)	3.72, m	3.05, m	3.11, m			3.28, m	3.12, m
24	2.44, m; 2.38, m	4.60 d (9.6)	3.98 dd (9.6)	2.37, m	2.43, m; 2.31, m	2.21, t (7.8)	7.43, d (7.8)	7.55, m	2.25, m; 2.18, m	2.61, m; 2.33, m
26	1.10, s	1.56, s	1.45, s	1.48, s	1.48, s	1.48, s	1.53, s	1.52, s	1.40, s	1.53, s
27	1.09, s	1.54, s	1.37, s	1.46, s	1.46, s	1.46, s	1.53, s	1.52, s	1.40, s	1.51, s
28	1.73, s	1.28, s	1.30, s	1.53, s	1.65, s	1.38, s	1.36, s	1.55, s	1.58, s	1.60, s
29	1.16, s	1.35, s	1.33, s	1.33, s	1.38, s	1.41, s	1.40, s	1.31, s	1.30, s	1.29, s
30	1.44, s	1.51, s	1.50, s	1.47, s	1.48, s	1.51, s	1.51, s	1.63, s	1.61, s	1.61, s
OAc-16		2.17, s				2.13, s	2.08, s	2.06, s		
OAc-25			2.20, s	1.88, s	1.87, s	1.91, s	1.91, s			1.92, s
OCH_3_-24			3.62, s

**Table 2:** ^13^C NMR Spectroscopic Data (150 MHz, in Pyridine-*d*_5_) for Compounds **1**–**10**.

No.	1	2	3	4	5	6	7	8	9	10
1	35.2	35.1	35.1	32.0	34.2	35.8	34.2	30.5	30.5	30.6
2	71.2	71.4	71.4	71.7	70.6	70.5	70.5	73.3	73.3	73.3
3	81.9	80.8	81.8	82.1	80.8	80.8	80.8	78.9	79.0	79.0
4	43.2	43.3	43.1	42.8	44.8	44.8	44.8	46.2	46.2	46.2
5	142.3	142.9	142.9	145.7	168.3	168.5	168.5	73.9	74.0	74.0
6	119.0	119.0	119.0	118.1	124.9	124.8	124.8	131.7	131.4	131.4
7	24.8	24.4	24.4	24.4	200.2	199.7	199.7	130.4	131.0	131.0
8	44.4	43.0	43.0	42.5	59.3	58.6	58.6	50.0	50.6	50.6
9	48.5	48.6	48.4	39.9	51.1	48.2	48.2	49.4	50.2	50.2
10	34.7	34.8	34.8	35.0	36.8	36.8	36.8	35.6	35.7	35.7
11	213.7	213.0	213.2	77.0	211.5	211.0	211.0	214.7	215.4	215.5
12	49.8	49.1	49.1	41.2	49.7	49.4	49.4	51.2	51.4	51.4
13	48.7	49.3	49.5	49.2	49.6	49.4	49.4	48.2	48.6	48.6
14	54.3	50.9	51.0	49.6	48.9	50.6	50.7	49.8	50.3	50.3
15	47.2	44.2	44.4	46.2	47.1	44.5	44.5	44.0	46.8	46.8
16	56.8	75.1	75.2	70.6	70.7	74.9	74.6	75.1	71.1	71.1
17	121.8	55.2	56.0	59.4	59.4	56.0	56.2	58.6	59.3	59.6
18	21.4	20.6	20.5	19.7	20.1	20.5	20.6	20.5	20.6	19.2
19	21.0	20.7	20.8	26.2	21.5	21.5	21.5	26.7	23.0	23.1
20	146.6	80.3	80.6	80.4	80.5	80.3	80.0	79.2	80.5	80.5
21	15.0	25.2	25.9	25.5	26.0	25.7	25.3	23.0	22.0	25.9
22	200.4	215.4	216.2	215.3	215.4	216.4	204.5	204.5	216.4	215.4
23	71.7	40.8	40.0	39.3	32.7	33.2	121.6	120.3	33.1	32.6
24	51.0	75.0	85.0	35.3	35.8	35.8	151.9	157.2	38.9	35.8
25	79.0	72.5	72.5	81.4	82.0	81.7	80.2	70.7	69.5	82.0
26	28.4	28.2	28.3	25.7	26.0	26.2	27.0	30.4	30.3	26.5
27	28.5	26.0	25.3	25.8	26.4	26.5	26.7	30.3	30.3	26.4
28	20.2	19.4	19.3	20.0	20.1	19.7	19.7	24.8	21.3	22.0
29	22.6	23.0	22.9	21.4	23.5	23.5	22.1	19.3	19.3	20.7
30	25.9	24.4	26.0	25.8	25.1	25.2	25.2	21.5	25.8	21.3
OAc-16		171.0/21.6				170.9/21.6	170.8/21.7	170.7/21.4		
OAc-25				169.9/22.0	170.6/22.6	170.6/22.6	170.2/22.1			170.6/22.7
OCH_3_-24			60.5							

Compound **4** was isolated as a white powder. Its molecular formula was C_32_H_52_O_8,_ as inferred from the HR-ESIMS (*m/z* 587.3577 [M + Na]^+^, calculated for C_33_H_52_O_9_Na, 587.3560). The ^1^H NMR and ^13^C APT data of **4** were comparable with those of **2** (Tables 1 and 2). A comparison of **4** and **2** demonstrated that the carbon signals at C-16 (*δ*_C_ 70.6), C-11 (*δ*_C_ 77.0), and C-24 (*δ*_C_ 35.3) were shifted upfield, while the signal at C-25 (*δ*_C_ 81.4) was shifted downfield. These differences suggested that in **4**, the carbonyl group at C-11 in **2** was substituted by a hydroxyl group, the acetyl group at C-16 and hydroxyl group at C-25 found in **2** exchanged their positions and the methoxyl group at C-24 was absent. The determination of the relative configurations of the methyl groups and other protons in the tetracyclic ring based on the significant NOE correlation between H-3 (*δ*_H_ 3.47) and H_3_-29 (*δ*_H_ 1.33), H-11 and CH_3_-19 revealed their *β*-orientation. Considering the identical biogenetic of cucurbitacin triterpenes, the absolute configuration of C-11 was *R*. Thus, compound **4** was identified and was called hemsleyacin D.

Compound **5** was isolated as a white powder and corresponded to the molecular formula C_32_H_48_O_9_ based on the pseudomolecular ion at *m/z* 599.3196 (calculated for C_32_H_48_O_9_Na [M + Na]^+^, 599.3212), indicating nine degrees of unsaturation. The IR spectrum showed absorption bands corresponding to hydroxy (3411 cm^-1^) and unsaturated carbonyl (1719 cm^−1^) groups. The ^1^H and ^13^C APT spectra (Tables 1 and 2) of **5** were similar to those of **4** but differed in that the protons at C-7 and hydroxyl group at C-11 in **4** were all oxidized to carbonyl groups in **5**. The characteristic carbon signals in the ^13^C APT spectrum at C-7 (*δ*_C_ 200.2) and C-11 (*δ*_C_ 211.5) in combination with the HMBC correlations from H-6 (*δ*_H_ 6.63, s) to and H_3_−19 (*δ*_H_ 1.27, s) to C-8 (*δ*_C_ 59.3) supported the above result. Based on this, the structure of compound **5** was determined and it was called hemsleyacin E.

Compound **6** possessed a molecular formula of C_34_H_50_O_10_, as indicated by the HR-ESIMS and NMR examinations. The ^1^H and ^13^C NMR (Tables 1 and 2) data of **6** were similar to those of compound **5**, even though the hydroxyl group at C-16 found in **5** was replaced by an acetyl group in **6**. The extra NMR signals at *δ*_H_ 2.13, and *δ*_C_ 21.6 and 170.9 confirmed the existence of an additional acetyl group, while the HMBC correlations (Fig. 2) from H-16 to *δ*_C_ (170.9) supported the position of the acetyl group at C-16. Therefore, compound **6** was identified and was called hemsleyacin F.

Compound **7** was separated as a white powder. Its molecular formula was determined to be C_34_H_48_O_10_ based on HR-ESIMS (*m/z* 639.7270 [M + Na]^+^, calculated for C_30_H_44_O_6_Na, 639.7287). An evaluation of the NMR patterns of **7** (Tables 1 and 2) and **6** demonstrated that **7** possessed a structure similar to **6**, with the exception of the signals corresponding to a double bond at *δ*_H_ 3.03 (H_1_-23), *δ*_H_ 3.11 (H_2_-23), *δ*_H_ 2.43 (H_2_-24), *δ*_H_ 2.31 (H_2_-24), *δ*_C_ 151.9 (C-24), and *δ*_C_ 121.6 (C-23) and the upfield chemical shift of carbonyl carbon at C-22 (*δ*_C_ 204.5). These differences suggested the existence of a double bond between C-23 and C-24 that was conjugated with the carbonyl carbon at C-22. The HMBC correlations (Fig. 2) from C-16 to C-23 validated the above result. Compound **7** was thus identified and was named hemsleyacin G.

Compound **8** had a pseudomolecular ion peak at *m/z* 599.3143 (calculated for C_32_H_48_O_9_Na, 599.3196) [M + Na]^+^ in the positive ion HR-ESIMS with a molecular formula of C_32_H_48_O_9_. The ^1^H and ^13^C APT spectroscopic data (Tables 1 and 2) of **8** matched those of **7**, except for the rearrangement of an *α*, *β*-unsaturated ketone at C-5/C-6/C-7. The olefinic data of H–6 (*δ*_H_ 6.37, d, *J* = 9.0 Hz) and H-7 (*δ*_H_ 5.65, m), C-6 (*δ*_C_ 131.7) and C-7 (*δ*_C_ 130.4) suggested that the carbonyl group at C-7 in **7** was reduced and dehydrated and formed this double bond in **8**. The downfield chemical shift of C-5 (*δ*_C_ 73.9) in combination with the molecular formula C_32_H_48_O_9_ revealed that the double bond at C-5/C-6 in **7** was also reduced, and one hydroxyl group was placed at C-5 in **8**. The HMBC correlations (Fig. 2) confirmed this deduction. Considering the biogenetic relationship of A/B trans ring, the hydroxyl group at C-5 was established as *β*-oriented. Thus, the structural characteristics of **8** were determined and the compound was called hemsleyacin H.

Compound **9** was shown to possess a molecular formula of C_30_H_48_O_8_ based on the HRESIMS ion peak at *m*/*z* 559.3253 (calculated for C_30_H_48_O_8_Na [M + Na]^+^, 559.3247) and an examination of ^13^C APT data, and had two fewer degrees of unsaturation than **8**. A comparison of the NMR spectra of **9** with that of **8** indicated that their structures were similar, except that the carbon signals of C-16 (*δ*_C_ 71.1), C-23 (*δ*_C_ 33.1), and C-24 (*δ*_C_ 38.9) in **9** were shifted upfield. These differences suggested that the double bond at C-23/C-24 in **8** was absent in **9** and that at C-16, a hydroxyl group was present in **9** instead of the acetyl group in **8**. In the HMBC spectrum, the correlation between the proton signals of H_2_-24 (*δ*_H_ 2.18) and C-22 (*δ*_C_ 216.2), and H_2_-23 (*δ*_H_ 3.28) and C-25 (*δ*_C_ 72.5) confirmed the lack of a double bond at C-23/C-24 in **9**. The relative configuration of **9** was deduced by the NOESY spectrum. Based on these results, compound **9** was named hemsleyacin I.

Compound **10** was purified as a white powder with a molecular formula of C_32_H_50_O_9_ based on the HR-ESIMS ion peak at *m/z* 601.3328 [M + Na]^+^ (calculated for C_32_H_50_O_9_Na, 601.3353). The NMR spectroscopic data of **10** closely matched those of **9**, except that the appearance of an additional acetyl signal (*δ*_C_ 82.0, C-25). C-25 (*δ*_C_ 82.0) was downfield when compared with C-25 (*δ*_C_ 69.5) of **9**, suggesting that the hydroxyl group at C-25 in **9** was substituted by a single acetyl group in **10** in the ^13^C APT NMR spectrum. The HMBC correlations (Fig. 2) from H_3_-26 (*δ*_H_ 1.53, s) to *δ*_C_ 170.6 together with the molecular formula confirmed the above deduction. Thus, compound **10** was identified and named hemsleyacin J.

**Figure 5:**
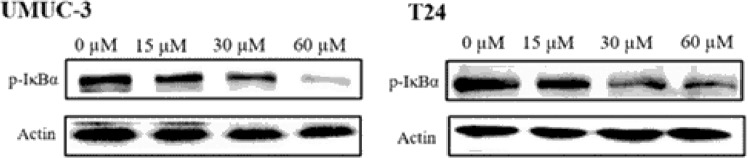
Western blot analysis of dihydrocucurbitacin F.

The antifeedant activities of compounds **7** and **10-12** were evaluated against *Plutella xylostella* larvae. The result showed that compound **10** exhibited potent antifeedant activity against *P*. *xylostella* larvae after 48 h of treatment with the highest antifeedant rate of 43.57% at the concentration of 0.5% (Table 3). Furthermore, the cytotoxicity of all of the isolated compounds was evaluated against the UMUC-3 and T24 bladder cancer cell lines according to the MTT procedure. The results (Table 4) showed that compound **11** exhibited potent cytotoxic activity against the UMUC-3 and T24 cell lines with IC_50_ values of 29.12 and 35.62 *μ*M, respectively, while the other compounds displayed moderate effects with the IC_50_ values of 62.34–97.15 *μ*M compared with the positive control cisplatin (IC_50_ values of 8.27 and 13.72 *μ*M). To assess whether the inhibitory activity of dihydrocucurbitacin F on bladder cancer cells was associated with the cessation of the NFκB pathway, the phosphorylation of IκBα in T24 and UMUC-3 cells was detected by western blot analysis with p-IκBα-specific antibodies after the cells were exposed to dihydrocucurbitacin F for 48 h. The results revealed that dihydrocucurbitacin F treatment substantially inhibited the phosphorylation of IκBα (Fig. 5).

**Table 3:** Antifeeding activity of the tested compounds from *Plutellaxylostell alarvae*.

Conc. (c%)	Antifeedant rate (%)				
No.	0.5	0.25	0.1	0.05	0.01
**7**	38.00	36.31	32.66	30.59	27.67
**10**	43.57	41.79	38.97	35.83	33.06
**11**	33.88	31.93	31.23	30.18	29.23
**12**	35.31	33.76	33.20	30.95	26.56

**Table 4:** IC_50_ values of compounds **1**–**13** against human cancer cells.

Compounds	IC_50_ (μM)	
	UMUC-3	T24
**1**	75.62 ± 2.3^a^	89.1 ± 6.3
**2**	72.59 ± 3.6	75.47 + 4.2
**3**	65.71 ± 2.9	92.23 ± 5.7
**4**	62.34 ± 2.7	73.63 ± 3.8
**5**	69.00 ± 4.6	81.28 ± 5.2
**6**	64.45 ± 3.7	88.67 ± 4.8
**7**	74.20 ± 4.4	88.56 ± 6.2
**8**	68.66 ± 3.4	97.15 ± 7.6
**9**	69.21 ± 6.1	84.27 ± 7.1
**10**	75.89 ± 3.3	79.82 ± 4.7
**11**	29.12 ± 2.0	35.62 ± 3.3
**12**	78.70 ± 4.6	96.14 ± 5.9
**13**	63.86 ± 3.0	84.25 ± 6.1
**cisplatin** ^**b**^	8.27 ± 1.9	13.72 ± 2.4

^a^Value presents the mean ± SD of triplicate experiments.

^b^Positive control substance.

## Methods

### Experimental procedures

IR spectra and UV absorption spectra were measured using FTIR-8400S and Shimadzu UV2550 spectrometers, respectively. Optical rotations were determined using a Jasco P-1010 polarimeter. TNMR spectra were recorded with a Bruker AV III 600 NMR spectrometer using trimethylsilyl (TMS) as the internal standard. HR-ESIMS spectra were obtained using a LTQ-Obitrap XL spectrometer. Preparative HPLC was performed using a Shimadzu analytic LC furnished with two LC-6AD pumps, a SPD-20A UV detector, and a Venusil C18 column (250 mm × 10 mm, i.d., 5 μM, Agela Technologies Ltd., the U.S.) Elution was carried out with CH_3_OH-H_2_O at a flow rate of 2 mL/min. C18 reversed-phase silica gel (40~63 μM, Merck, Darmstadt, Germany), MCI gel (CHP 120 P, 75~150 μM, Mitsubishi, Japan), and silica gel (SiO_2_ 100~200 mesh, Qingdao Marine Chemical Plant, Qingdao, PRC) were used for column chromatography. GF254 precoated silica gel plates were used for thin-layer chromatography (TLC) VWR International Ltd. (Lutterworth, UK). Analytical grade solvents were used in all of the experiments (Beijing Chemical Works).

### Plant material

The tubers of *H*. *penxianensis* were obtained in 2014 in Chongqing, China (for GPS 29.0316099528,107.1971212986). Identification was carried out by Professor Yi Sirong (The Institute of Alpine Economic Plant at the Yunnan Academy of Agricultural Sciences). Specimens were maintained at The Institute of Medicinal Plant Development (No. CS140921).

### Extraction and isolation

The air-dried ground tubers of *H*. *penxianensis* (total of 10.0 kg) were extracted with 95% ethanol (6 L) three times for 3 h each time. After the removal of the solvent, the crude extract (1.1 kg) was combined with H_2_O and then partitioned with petroleum ether (3 × 1 L), ethyl acetate (4 × 2 L), and *n*-butanol (3 × 2 L). The EtOAc fraction (200 g) was placed in silica gel (100–200 mesh, 8 × 100 cm) and eluted with CH_2_Cl_2_-MeOH (from 100:1 to 0:1), affording 12 fractions, namely, A–L. Fraction E (12.7 g) was eluted using MCI column chromatography with MeOH-H_2_O (3:7 → 1:0), giving eight fractions (Fr.E.1–E.8). Semipreparative HPLC (CH_3_CN-H_2_O, 85:15) was used for the purification of Fr.E.3 to give compound **2** (3.6 mg, *t*_R_ = 16.4 min), compound **3** (4.1 mg, *t*_R_ = 18.1 min), and compound **1** (3.1 mg, *t*_R_ = 22.7 min). Fraction I (18.3 g) was subjected to MPLC using MCI-gel column chromatography with MeOH-H_2_O (3:7 → 1:0, v/v) to afford eight subfractions (Fr.I.1–I.8). Fr.I.5 (5.2 g) was isolated for use in ODS column chromatography and eluted with MeOH-H_2_O (3:7 → 1:0, v/v), resulting in eight fractions (Fr. I.5.1–I.5.8). Further purification of Fr. I.5.6 (1.1 g) was achieved by semipreparative HPLC (CH_3_CN-H_2_O, 70:30), resulting in compound **6** (5.0 mg, *t*_R_ = 18.8 min) and compound **4** (3.5 mg, *t*_R_ = 25.4 min). Fr.I.5.7 (0.9 g) was chromatographed by semipreparative HPLC (CH_3_CN-H_2_O, 80:20), affording compound **7** (6.3 mg, *t*_R_ = 20.6 min) and compound **5** (3.6 mg, *t*_R_ = 23.9 min). Using ODS column chromatography elution, Fr.I.6 (4.4 g) was further separated with MeOH-H_2_O (3:7 → 1:0, v/v), giving eight fractions (Fr.I.6.1–I.6.8). Fr.I.6.6 (0.9 g) was chromatographed by semipreparative HPLC (CH_3_CN-H_2_O, 85:15), providing compound **9** (3.0 mg, *t*_R_ = 19.5 min), compound **10** (6.6 mg, *t*_R_ = 23.2 min), and compound **8** (4.1 mg, *t*_R_ = 27.2 min). Fr.I.6.7. (1.0 g) was purified using semipreparative HPLC (CH_3_CN-H_2_O, 75:25), providing compound **11** (12.1 mg, *t*_R_ = 18.2 min), **12** (5.3 mg, *t*_R_ = 27.6 min), and **13** (3.8 mg, *t*_R_ = 29.2 min).

*Hemsleyacin A* (**1**). White powder; [*α*]_20 D_ + 38 (*c* = 0.22, MeOH); UV (CH_3_OH) *λ*_max_ (log *ε*): 205 (4.12), 244 (3.19) nm; IR (KBr) *ν*_max_: 3384, 2955, 1724, 1619, 1338 cm^–1^; ^1^H NMR and ^13^C-APT see Tables 1 and 2; HRESIMS (+) *m/z* 522.3333 [M + Na]^+^ (calculated for C_30_H_44_O_6_Na, 523.3036).

*Hemsleyacin B* (**2**). White amorphous powder; [*α*]_20 D_ + 55 (*c* = 0.10, MeOH); UV (CH_3_OH) *λ*_max_ (log *ε*): 203 (3.98), 250 (2.70) nm; IR (KBr) *ν*_max_: 3370, 2931, 1692, 1652, 1369 cm^–1^; ^1^H NMR and ^13^C-APT see Tables 1 and 2; HR-ESIMS (+) *m/z*: 601.3369 [M + Na]^+^ (calculated for C_32_H_50_O_9_Na, 601.3353).

*Hemsleyacin C* (**3**). White amorphous powder; [*α*]_20 D_ + 12 (*c* = 0.38, MeOH); UV (CH_3_OH) *λ*_max_ (log *ε*): 202 (4.85), 250 (2.34) nm; IR (KBr) *ν*_max_: 3354, 2914, 1675, 1611, 1342 cm^–1^; ^1^H NMR and ^13^C-APT see Tables 1 and 2; HR-ESIMS (+) *m/z*: 615.3527 [M + Na]^+^ (calculated for C_33_H_52_O_9_Na, 615.3509).

*Hemsleyacin D* (**4**). White amorphous powder; [*α*]_20 D_ + 53 (*c* = 0.48, MeOH); UV (CH_3_OH) *λ*_max_ (log *ε*): 202 (4.68), 250 (2.17) nm; IR (KBr) *ν*_max_: 3367, 2873, 1689, 1631, 1282 cm^–1^; ^1^H NMR and ^13^C-APT see Tables 1 and 2; HR-ESIMS (+) *m/z*: 587.3577 [M + Na]^+^ (calculated for C_32_H_52_O_8_Na, 587.3560).

*Hemsleyacin E* (**5**). White amorphous powder; [*α*]_20 D_ + 32 (*c* = 0.39, MeOH); UV (CH_3_OH) *λ*_max_ (log *ε*): 202 (4.09), 245 (3.57) nm; IR (KBr) *ν*_max_: 3299, 2825, 1707, 1608, 1331 cm^–1^; ^1^H NMR and ^13^C-APT see Tables 1 and 2; HR-ESIMS (+) *m/z*: 599.3196 [M + Na]^+^ (calculated for C_32_H_48_O_9_Na, 599.3212).

*Hemsleyacin F* (**6**). White amorphous powder; [*α*]_20 D_ + 34 (*c* = 0.17, MeOH); UV (CH_3_OH) *λ*_max_ (log *ε*): 202 (4.22), 245 (3.68) nm; IR (KBr) *ν*_max_: 3396, 2934, 1697, 1605, 1328 cm^–1^; ^1^H NMR and ^13^C-APT see Tables 1 and 2; HR-ESIMS (+) *m/z*: 641.3322 [M + Na]^+^ (calculated for C_34_H_50_O_10_Na, 641.3302).

*Hemsleyacin G* (**7**). White amorphous powder; [*α*]_20 D_ + 26 (*c* = 0.28, MeOH); UV (CH_3_OH) *λ*_max_ (log *ε*): 205 (3.83), 244 (3.20) nm; IR (KBr) *ν*_max_: 3386, 2873, 1697, 1621, 1323 cm^–1^; For ^1^H NMR (600 MHz, pyridine-d_5_) and ^13^C-APT (150 MHz, pyridine-d_5_) spectroscopic data see Tables 1 and 2; HR-ESIMS (+) *m/z*: 639.3159 [M + Na]^+^ (calculated for C_30_H_44_O_6_Na, 639.3145).

*Hemsleyacin H* (**8**). White amorphous powder; [*α*]_20 D_ + 37 (*c* = 0.29, MeOH); UV (CH_3_OH) *λ*_max_ (log *ε*): 203 (4.56), 251 (3.04) nm; IR (KBr) *ν*_max_: 3372, 2922, 1712, 1620, 1335 cm^–1^; ^1^H NMR and ^13^C-APT see Tables 1 and 2; HR-ESIMS (+) *m/z*: 599.3143 [M + Na]^+^ (calculated for C_32_H_48_O_9_Na, 599.3196).

*Hemsleyacin I* (**9**). White amorphous powder; [*α*]_20 D_ + 38 (*c* = 0.23, MeOH); UV (CH_3_OH) *λ*_max_ (log *ε*): 202 (4.17), 250 (2.76) nm; IR (KBr) *ν*_max_: 3377, 2960, 1710, 1605, 1327 cm^–1^; ^1^H NMR and ^13^C-APT see Tables 1 and 2; HR-ESIMS (+) *m/z*: 559.3253 [M + Na]^+^ (calculated for C_30_H_48_O_8_Na, 559.3247).

*Hemsleyacin J* (**10**). White amorphous powder; [*α*]_20 D_ + 36 (*c* = 0.17, MeOH); UV (CH_3_OH) *λ*_max_ (log *ε*): 202 (4.10), 250 (2.85) nm; IR (KBr) *ν*_max_: 3367, 2946, 1708, 1618, 1329 cm^–1^; ^1^H NMR and ^13^C-APT see Tables 1 and 2; HR-ESIMS (+) *m/z*: 601.3328[M + Na]^+^ (calculated for C_32_H_50_O_9_Na, 601.3353).

### Antifeedant activity analysis

Compounds **7**, **10**–**12** (5 mg each, enough amount of these compounds only) were accurately weighed in 1 mL volumetric flasks and dissolved in distilled acetone (0.5 mL). The volume was then adjusted to 1 mL with acetone to give a 0.5% stock solution. The stock solution was diluted to different concentrations (0.5, 0.25, 0.1, 0.05, and 0.01%) with 0.5% emulsified water. Cabbage leaves of uniform thickness were selected, and leaf discs were prepared with a punch (d = 1.7 cm). The solution was evenly spread onto the front and back sides of the cabbage leaf discs. A Tween 80 aqueous solution containing φ = 0.2% with a small amount of ethanol was used as a control group. After the solvent was naturally volatilized, it was placed in a petri dish (d = 9 cm) padded with moist filter paper. Each disc has four larvae heads of *P*. *xylostella*, three times per treatment, and treated with 9 panels at 48 h after treatment. The panels were used to measure the area of the leaf eaten by the larvae of *P*. *xylostella*. The experiments were conducted at 28 ± 1 °C and 65 ± 5% relative humidity. Corrected feeding inhibition (%) was calculated based on the formula:

$${\rm{feeding}}\,{\rm{inhibition}}\,( \% )=[({\rm{C}}-{\rm{T}})/({\rm{C}}+{\rm{T}})]\times 100,$$

where T is the consumption of the leaf in the treatment and C is the consumption of the leaf in the control.

### Cytotoxicity assays

The cytotoxicities of the tested compounds (**1**–**13**) were determined using the MTT assay. The UMUC-3 and T24 cancer cells were cultivated in Dulbecco’s Modified Eagle Medium (DMEM) with 10% fetal bovine serum and cultured at a density of 1.2 × 104 cells/mL in a 96-well microtiter plate. Subsequently, five different concentrations of each compound, resuspended in dimethyl sulfoxide (DMSO), were placed into the wells. Three replicates of each concentration were tested. Following incubation at 5% CO_2_ at 37 °C for 48 h, 10 μL of MTT (4 mg/mL) was inserted into each well, following which the cells were incubated for a further 4 h. The liquid in each well was then extracted and replaced with DMSO (200 μL). Using a microplate reader, absorbance was measured at a wavelength of 570 nm.

### Western blot analysis

The total proteins of the UMUC-3 and T24 cells were extracted with ice-cold RIPA buffer combined with protease and phosphatase inhibitor cocktail (Cowin Biotech Co., Ltd., Beijing, China). Equal amounts of total cell lysates were boiled in Laemmli SDS sample buffer, separated by 10% SDS-PAGE and then transferred to polyvinylidene difluoride membranes (PVDF). Following blocking with 5% nonfat milk for 2 h, the membrane was probed with primary antibody at 4 °C overnight and then incubated with horseradish peroxidase (HRP)-conjugated secondary antibody. Using a commercial ECL kit (Cowin Biotech Co., Ltd), the target bands were visualized by enhanced chemiluminescence.

## Supplementary information

Supplementary Information
