# Supplementary material for: Polyhydroxy cucurbitane triterpenes from Hemsleya penxianensis tubers
Source: Sci Rep. 2019 Aug 14;9:11835. doi: 10.1038/s41598-019-48365-0 (PMC6694101; doi:10.1038/s41598-019-48365-0)
Supplement: Supplementary file 1 — Supplementary Information [file 41598_2019_48365_MOESM1_ESM.docx]

**Polyhydroxy cucurbitane triterpenes from *Hemsleya penxianensis* tubers**

**Zhaocui Sun,^1,a^ Meigeng Hu,^1,a^ Nailiang Zhu,^1^ Xiaowei Huo,^2^ Xiaolei Zhou, ^1^ Zhonghao Sun,^1^ Junshan Yang,^1^ Guoxu Ma,^[[1]](#footnote-1)^*^,1^ and Xudong Xu^*,1^**

^1^ Key Laboratory of Bioactive Substances and Resource Utilization of Chinese Herbal Medicine, Ministry of Education, Institute of Medicinal Plant Development, Peking Union Medical College and Chinese Academy of Medical Sciences, Beijing 100193, People’s Republic of China

^2^ College of Pharmaceutical Science, Key Laboratory of Pharmaceutical Quality Control of Hebei Province, Hebei University. Baoding 071002, China

**ABSTRACT**: Ten new cucurbitane triterpenoids, hemsleyacins A–J (**1**–**10**), together with three known cucurbitane triterpenoids, dihydrocucurbitacin F (**11**), scandenogenin D (**12**), and jinfushanencin F (**13**), were separated from ethanolic tuber extracts of *Hemsleya penxianensis*. The absolute configurations of the new compounds were established on the bais of NMR, HRESIMS, and ECD spectra. Compounds **7** and **10-12** were evaluated in terms of their antifeedant activity against *Plutella xylostella* larvae. The result showed that compound **10** exhibited potent antifeedant activity against *P. xylostella* larvae after 48 h of treatment. Furthermore, MTT test displayed that compound **11** exhibited potent inhibitions toward the UMUC-3 and T24 cell lines with IC50 values of 29.12 and 35.62 μM, respectively, in comparison to the positive control cisplatin IC_50_ values of 8.27 and 13.72 μM. The Western blot analysis revealed that compound **11** treatments substantially inhibited the phosphorylation of IκBα.

List of Figures S1-S13

Figure S1. The CD Spectrum of **1** in MeOH

Figure S2. 1H-NMR (600 MHz, Pyridine-*d*_5_) spectrum of the new compound **1**

Figure S3. 13C-APT (150 MHz, Pyridine-*d*_5_) spectrum of the new compound **1**

Figure S4. HSQC spectrum of the new compound **1**

Figure S5. HMBC spectrum of the new compound **1**

Figure S5A. The enlarged HMBC spectrum of the new compound **1**

Figure S6. 1H-1H COSY spectrum of the new compound **1**

Figure S7. NOE spectrum of the new compound **1**

Figure S8. HR-ESI-MS spectrum of the new compound **1**.

Figure S9. The CD Spectrum of **2** in MeOH

Figure S10. 1H-NMR (600 MHz, Pyridine-*d*_5_) spectrum of the new compound **2**

Figure S11. 13C-APT (150 MHz, Pyridine-*d*_5_) spectrum of the new compound **2**

Figure S12. HSQC spectrum of the new compound **2**

Figure S13. HMBC spectrum of the new compound **2**

Figure S14. 1H-1H COSY spectrum of the new compound **2**

Figure S15. NOE spectrum of the new compound **2**

Figure S16. HR-ESI-MS spectrum of the new compound **2.**

Figure S17. 1H-NMR (600 MHz, Pyridine-*d*_5_) spectrum of the new compound **3**

Figure S18. 13C-APT (150 MHz, Pyridine-*d*_5_) spectrum of the new compound **3**

Figure S19. HSQC spectrum of the new compound **3**

Figure S20. HMBC spectrum of the new compound **3**

Figure S21. 1H-1H COSY spectrum of the new compound **3**

Figure S22. HR-ESI-MS spectrum of the new compound **3**.

Figure S23. 1H-NMR (600 MHz, Pyridine-*d*_5_) spectrum of the new compound **4**

Figure S24. 13C-APT (150 MHz, Pyridine-*d*_5_) spectrum of the new compound **4**

Figure S25. HSQC spectrum of the new compound **4**

Figure S26. HMBC spectrum of the new compound **4**

Figure S27. 1H-1H COSY spectrum of the new compound **4**

Figure S28. 1H-NMR (600 MHz, Pyridine-*d*_5_) spectrum of the new compound **5**

Figure S29. 13C-APT (150 MHz, Pyridine-*d*_5_) spectrum of the new compound **5**

Figure S30. HSQC spectrum of the new compound **5**

Figure S31. HMBC spectrum of the new compound **5**

Figure S32. 1H-1H COSY spectrum of the new compound **5**

Figure S33. HR-ESI-MS spectrum of the new compound **5**.

Figure S34. 1H-NMR (600 MHz, Pyridine-*d*_5_) spectrum of the new compound **6**

Figure S35. 13C-APT (150 MHz, Pyridine-*d*_5_) spectrum of the new compound **6**

Figure S36. HSQC spectrum of the new compound **6**

Figure S37. HMBC spectrum of the new compound **6**

Figure S38. 1H-1H COSY spectrum of the new compound **6**

Figure S39. HR-ESI-MS spectrum of the new compound **6**.

Figure S40. 1H-NMR (600 MHz, Pyridine-*d*_5_) spectrum of the new compound **7**

Figure S41. 13C-APT (150 MHz, Pyridine-*d*_5_) spectrum of the new compound **7**

Figure S42. HSQC spectrum of the new compound **7**

Figure S43. HMBC spectrum of the new compound **7**

Figure S44. 1H-1H COSY spectrum of the new compound **7**

Figure S45. HR-ESI-MS spectrum of the new compound **7**.

Figure S46. 1H-NMR (600 MHz, Pyridine-*d*_5_) spectrum of the new compound **8**

Figure S47. 13C-APT (150 MHz, Pyridine-*d*_5_) spectrum of the new compound **8**

Figure S48. HSQC spectrum of the new compound **8**

Figure S49. HMBC spectrum of the new compound **8**

Figure S50. 1H-1H COSY spectrum of the new compound **8**

Figure S51. HR-ESI-MS spectrum of the new compound **8**.

Figure S52. 1H-NMR (600 MHz, Pyridine-*d*_5_) spectrum of the new compound **9**

Figure S53. 13C-APT (150 MHz, Pyridine-*d*_5_) spectrum of the new compound **9**

Figure S54. HSQC spectrum of the new compound **9**

Figure S55. HMBC spectrum of the new compound **9**

Figure S56. 1H-1H COSY spectrum of the new compound **9**

Figure S57. HR-ESI-MS spectrum of the new compound **9**.

Figure S58. 1H-NMR (600 MHz, Pyridine-*d*_5_) spectrum of the new compound **10**

Figure S59. 13C-APT (150 MHz, Pyridine-*d*_5_) spectrum of the new compound **10**

Figure S60. HSQC spectrum of the new compound **10**

Figure S61. HMBC spectrum of the new compound **10**

Figure S62. HR-ESI-MS spectrum of the new compound **10**.


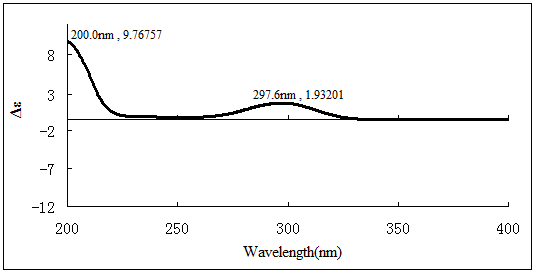


Figure S1. The CD Spectrum of **1** in MeOH.

Figure S2. ^1^H-NMR (600 MHz, pyridine-*d*_5_) spectrum of the new compound **1**

Figure S3. ^13^C-APT (150 MHz, pyridine-*d*_5_) spectrum of the new compound **1**


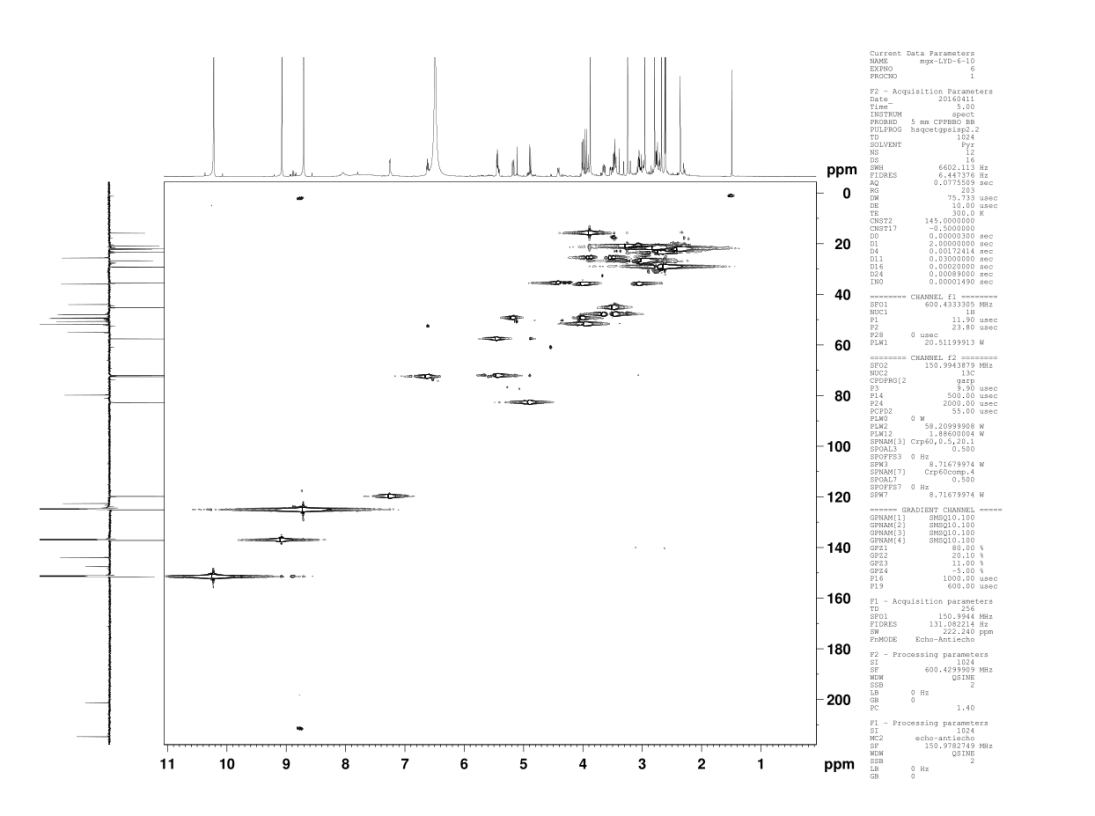


Figure S4. HSQC spectrum of the new compound **1**


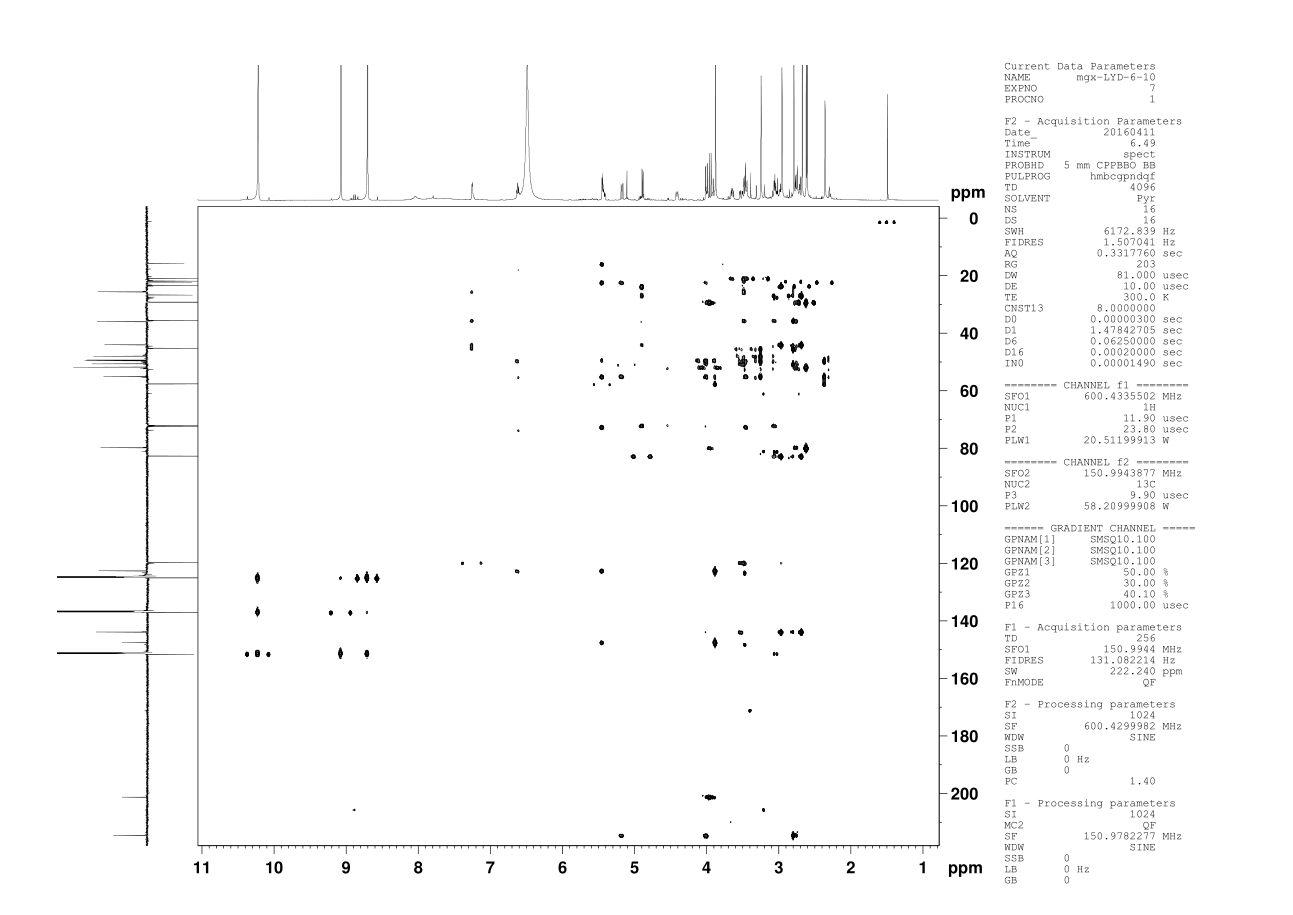


Figure S5. HMBC spectrum of the new compound **1**


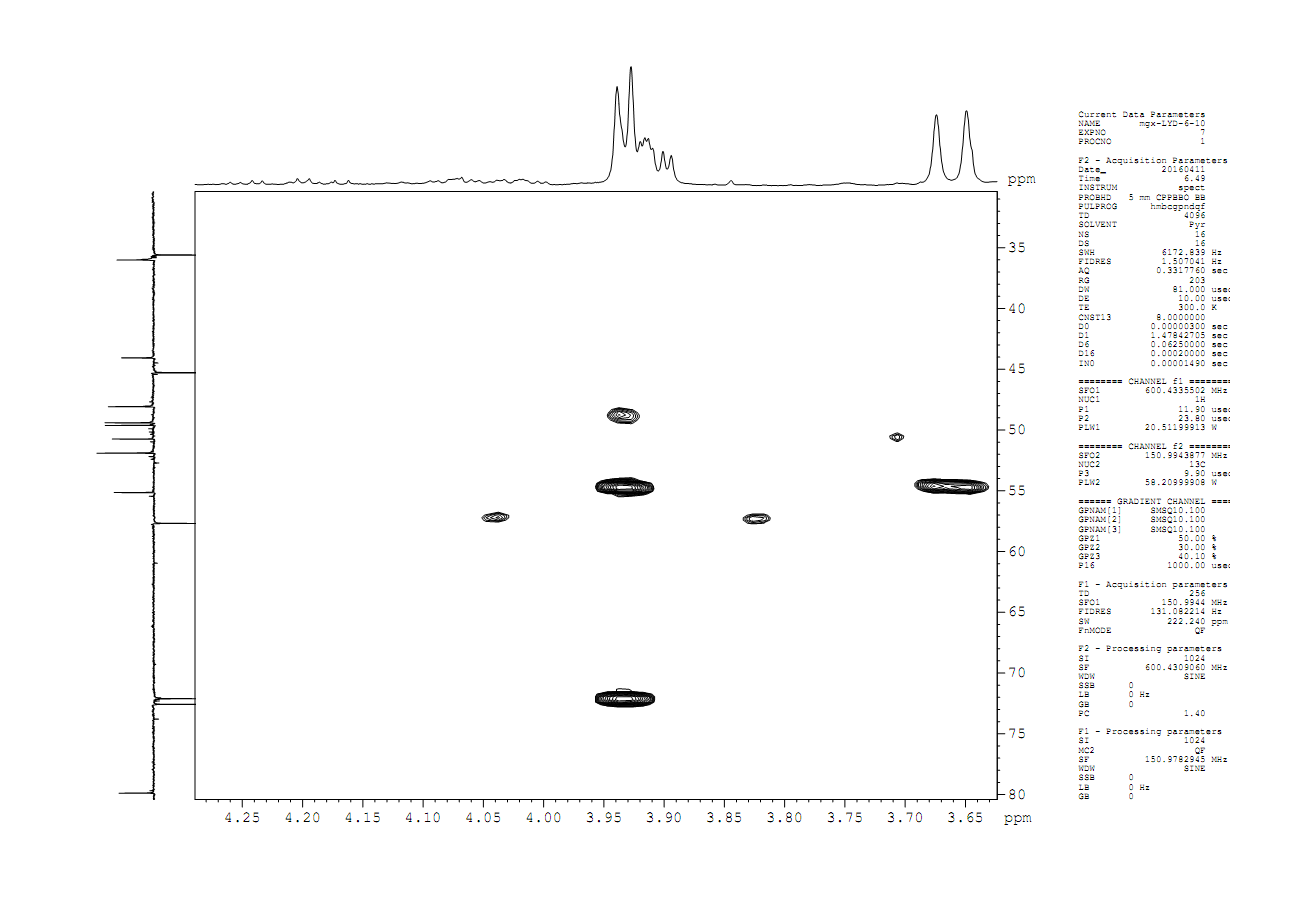
Figure S5A. The enlarged HMBC spectrum of the new compound **1**


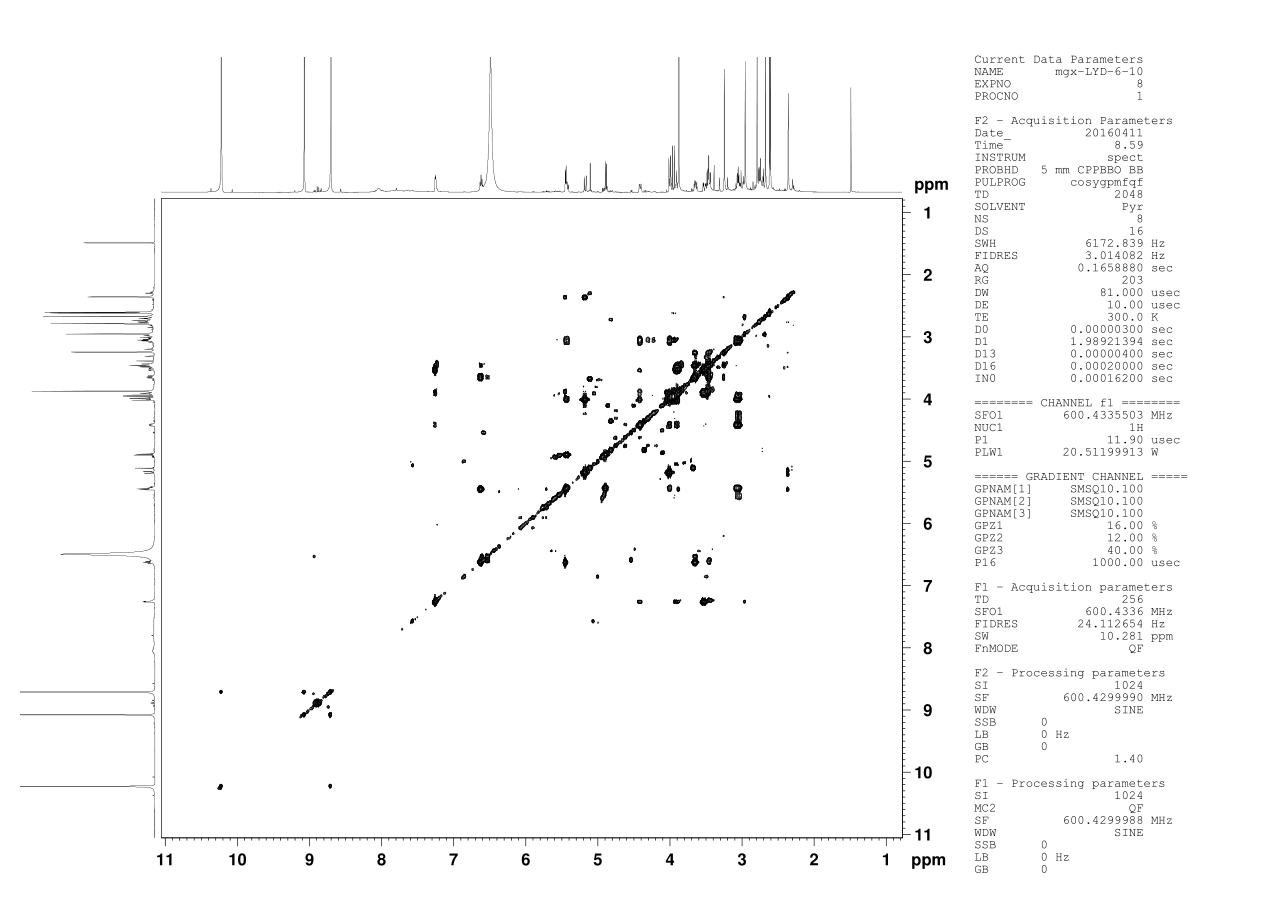


Figure S6. ^1^H-^1^H COSY spectrum of the new compound **1**


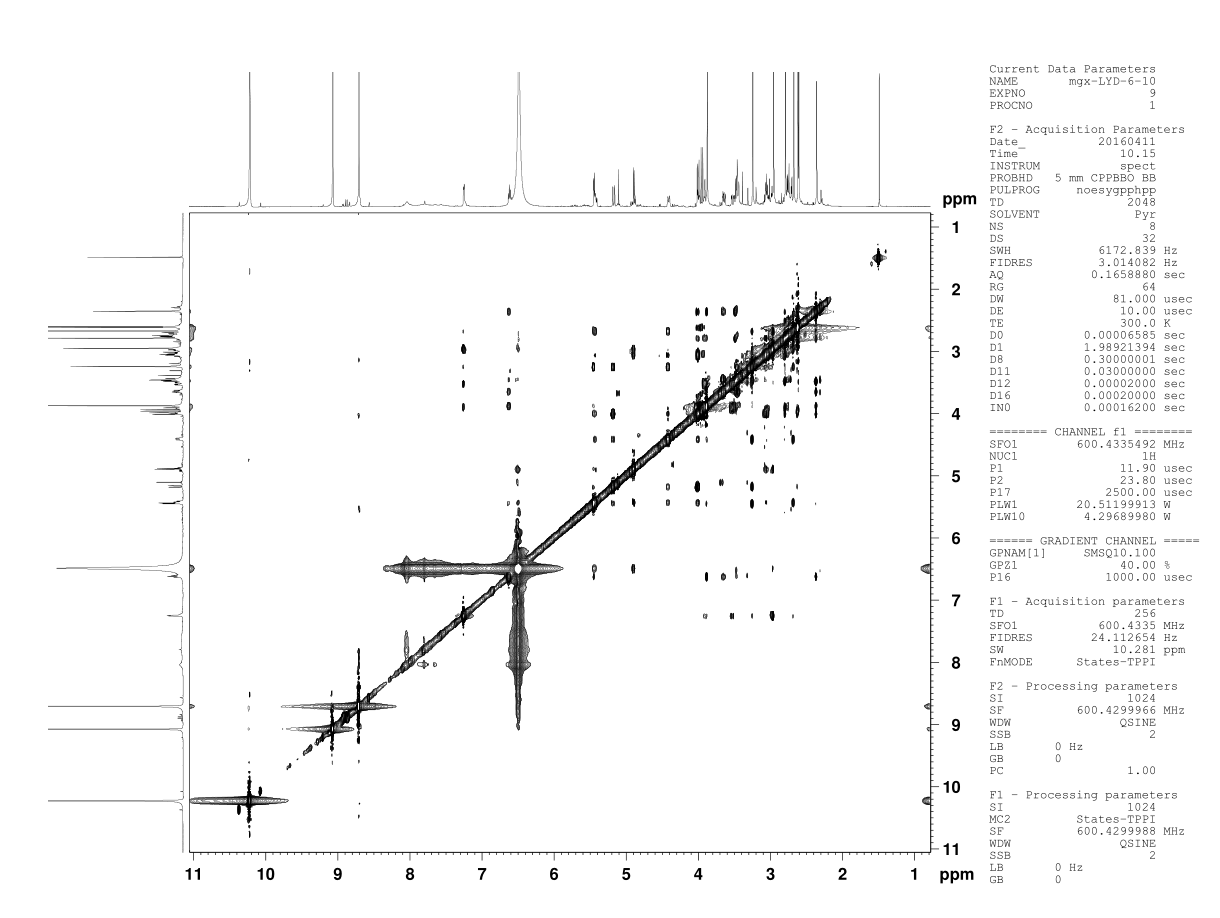


Figure S7. NOE spectrum of the new compound **1.**

Figure S8. HR-ESI-MS spectrum of the new compound **1**.


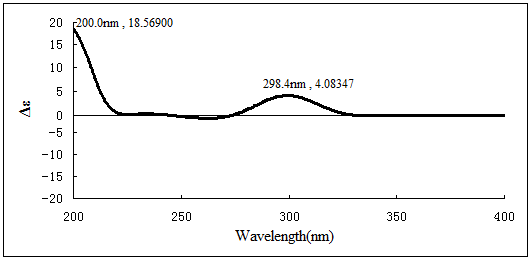


Figure S9. The CD Spectrum of **2** in MeOH.


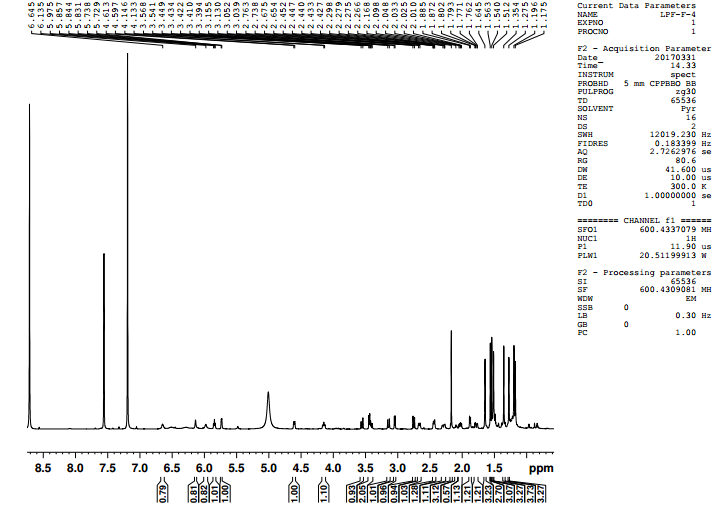


Figure S10. ^1^H-NMR (600 MHz, pyridine-*d_5_*) spectrum of the new compound **2**


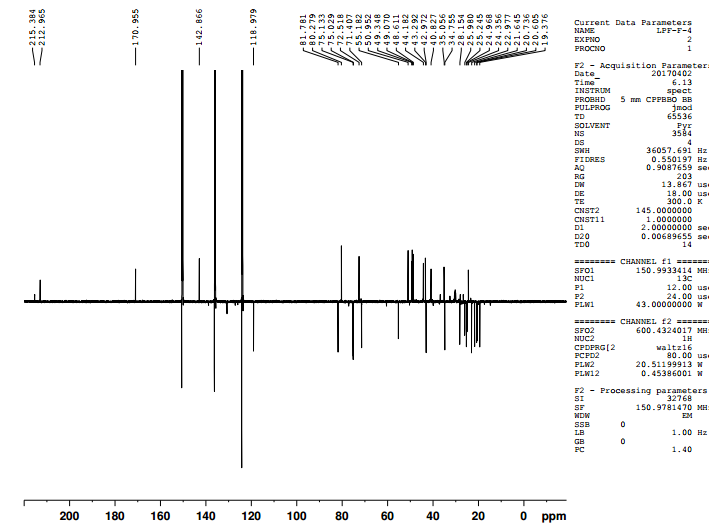


Figure S11. ^13^C-APT (150 MHz, pyridine-*d_5_*) spectrum of the new compound **2**


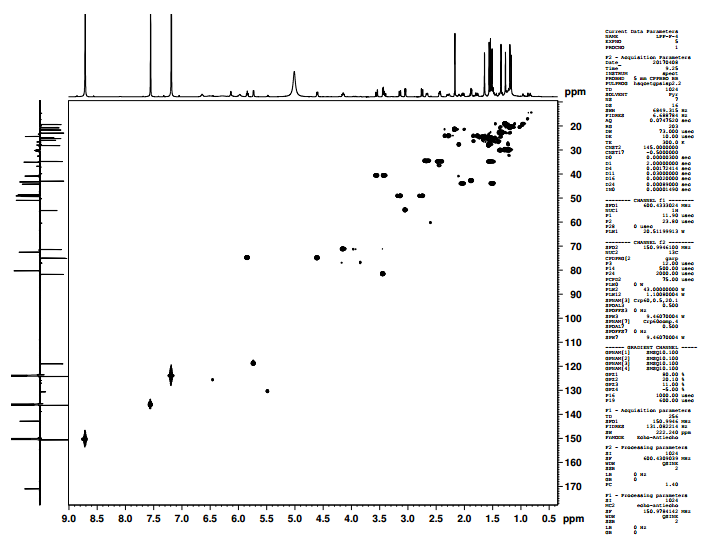


Figure S12. HSQC spectrum of the new compound **2**


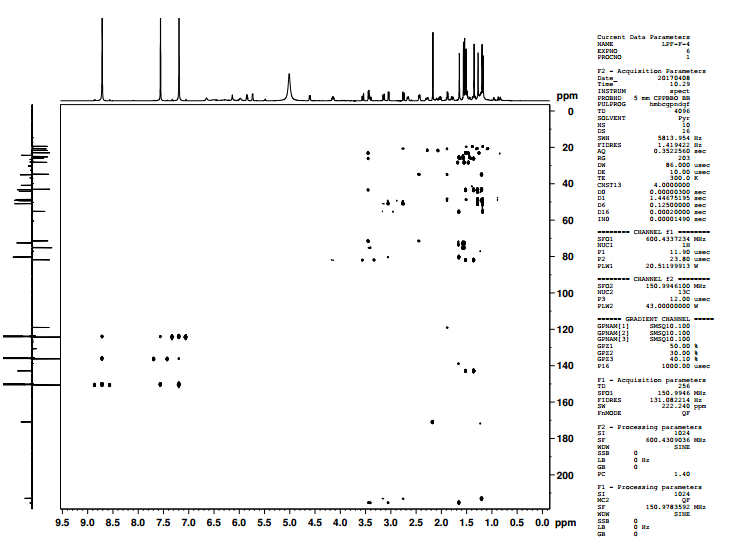


Figure S13. HMBC spectrum of the new compound **2**


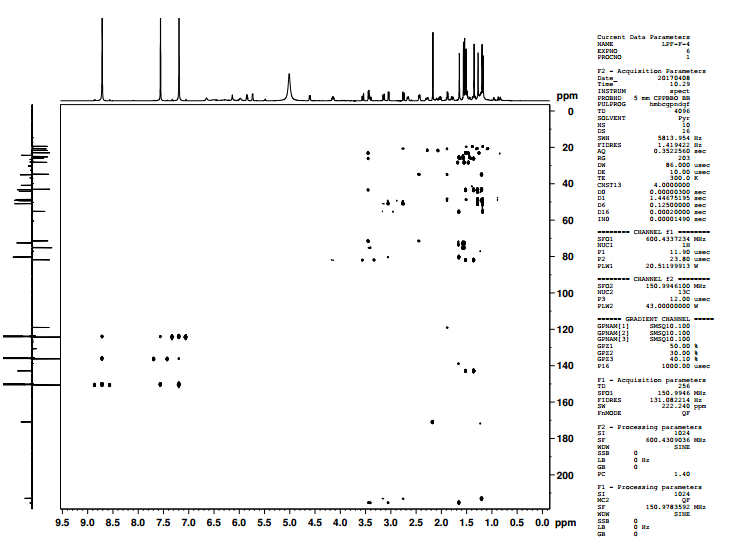


Figure S14.^1^H-^1^H COSY spectrum of the new compound **2**


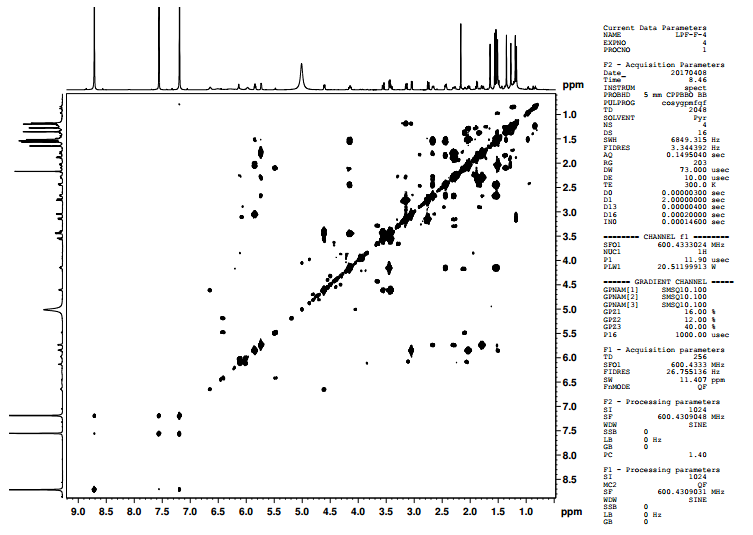


Figure S15. NOE spectrum of the new compound **2.**

Figure S16. HR-ESI-MS spectrum of the new compound **2**.


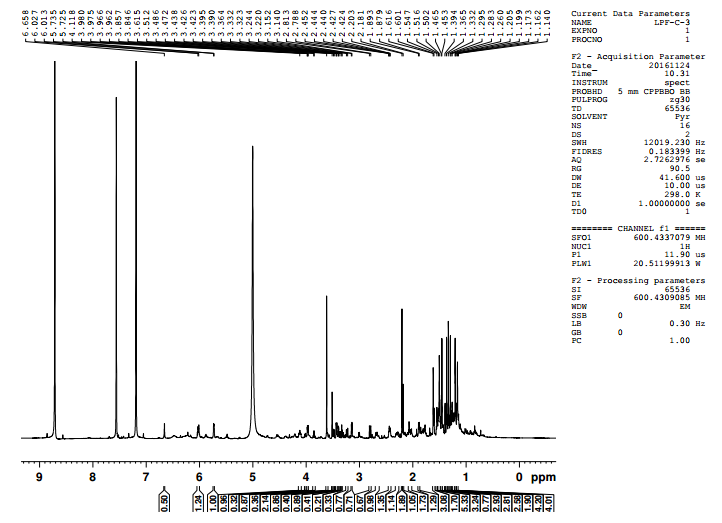


Figure S17. ^1^H-NMR (600 MHz, pyridine-*d_5_*) spectrum of the new compound **3**


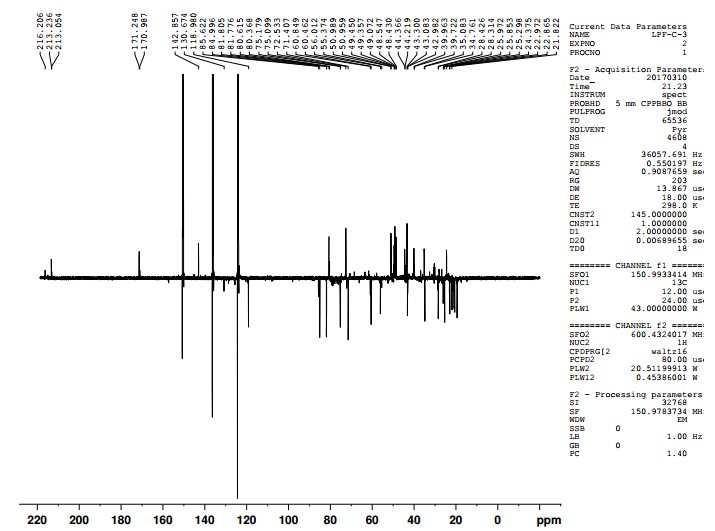


Figure S18. ^13^C-APT (150 MHz, pyridine-*d_5_*) spectrum of the new compound **3**


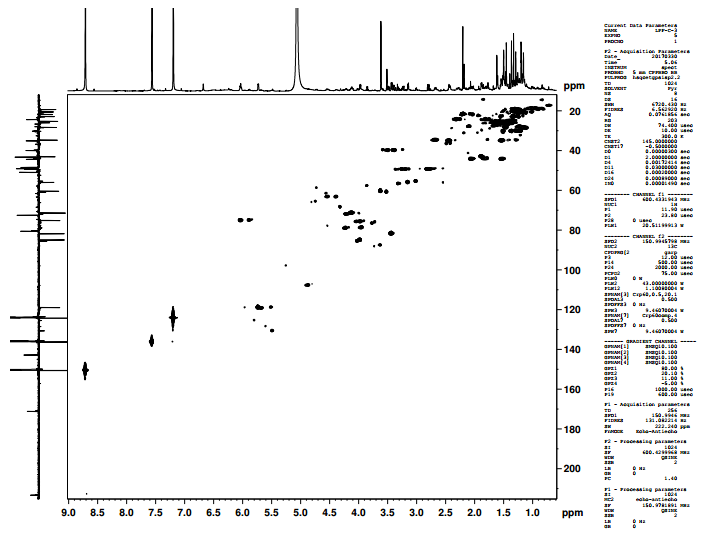


Figure S19. HSQC spectrum of the new compound **3**


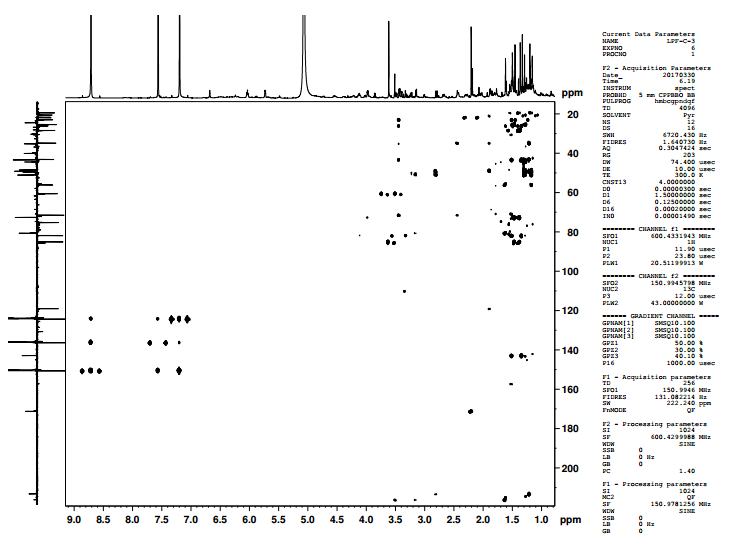


Figure S20. HMBC spectrum of the new compound **3**

Figure S21. HR-ESI-MS spectrum of the new compound **3**.


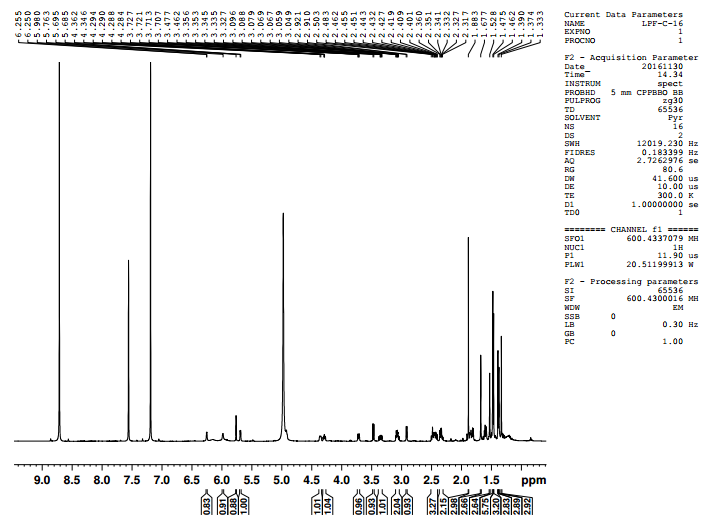


Figure S22. ^1^H-NMR (600 MHz, pyridine-*d_5_*) spectrum of the new compound **4**


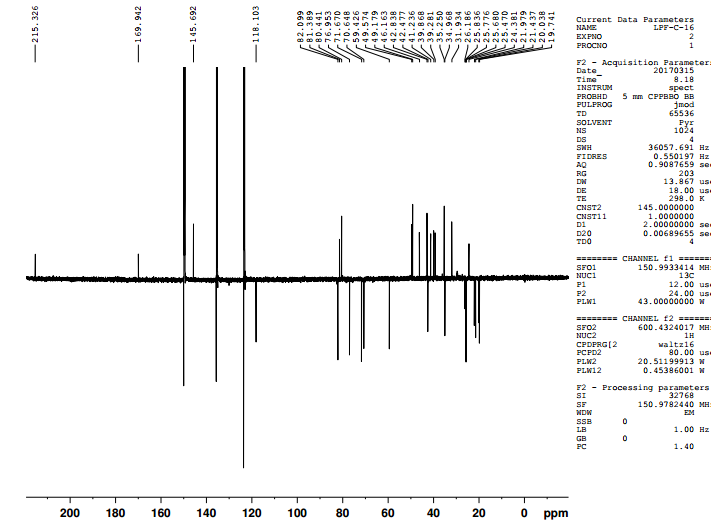


Figure S23. ^13^C-APT (150 MHz, pyridine-*d_5_*) spectrum of the new compound **4**


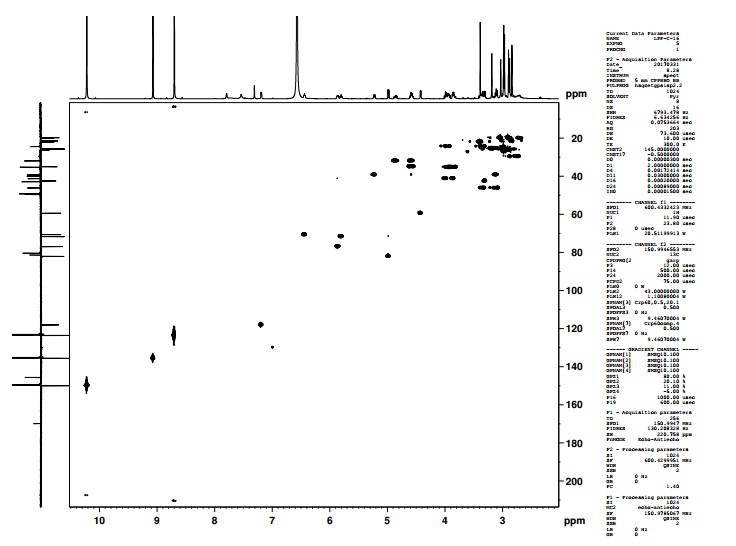


Figure S24. HSQC spectrum of the new compound **4**


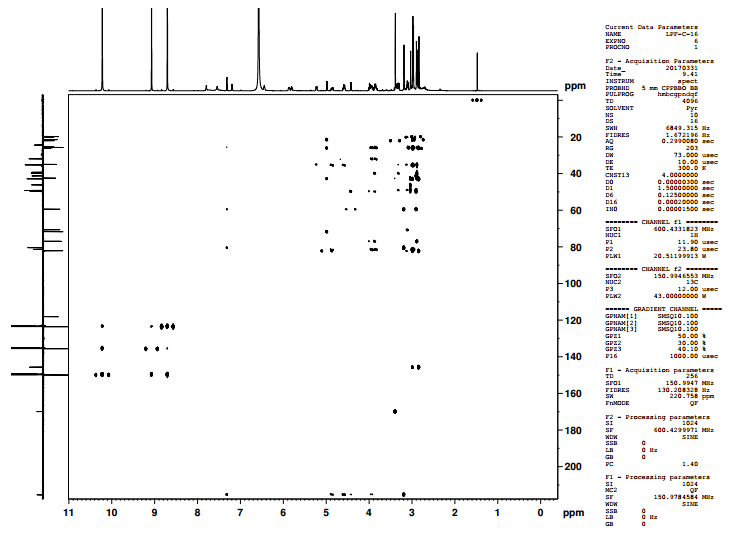


Figure S25. HMBC spectrum of the new compound **4**


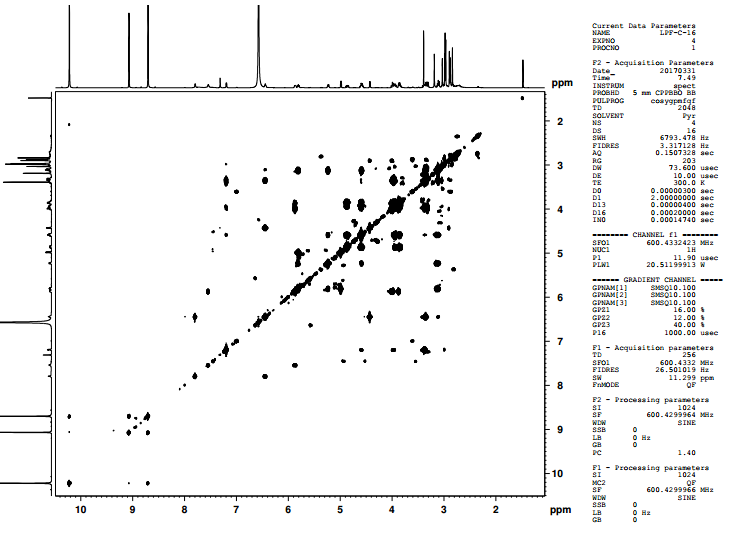


Figure S26 ^1^H-^1^H COSY spectrum of the new compound **4**

Figure S27. HR-ESI-MS spectrum of the new compound **4**.


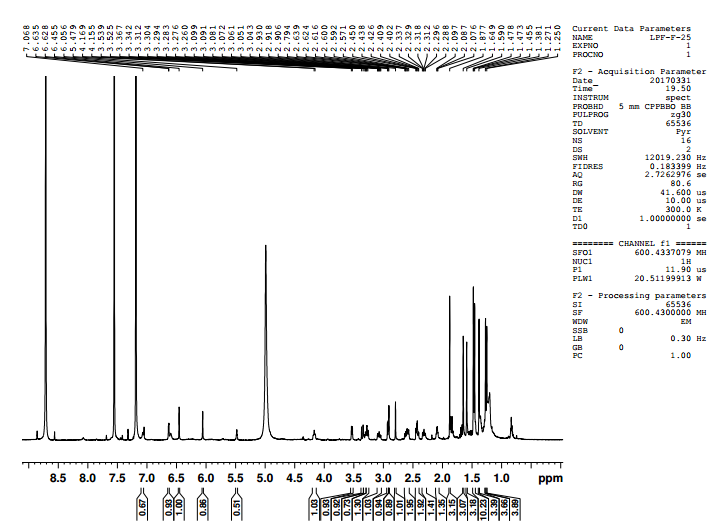


Figure S28. ^1^H-NMR (600 MHz, pyridine-*d_5_*) spectrum of the new compound **5**


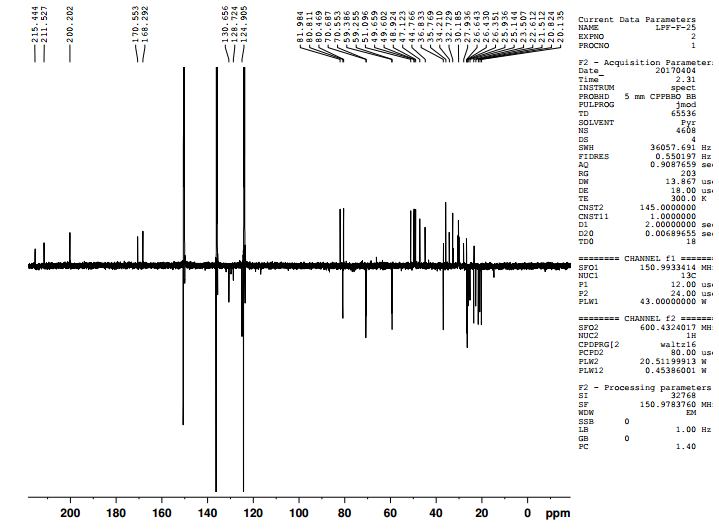


Figure S29. ^13^C-APT (150 MHz, pyridine-*d_5_*) spectrum of the new compound **5**


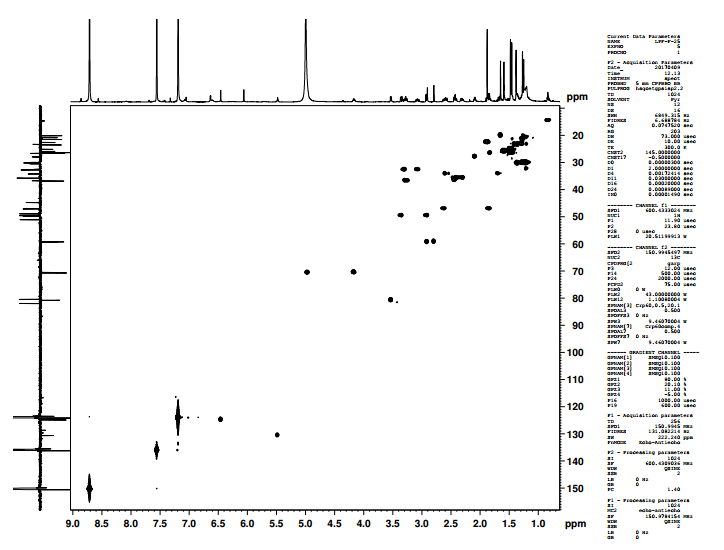


Figure S30. HSQC spectrum of the new compound **5**


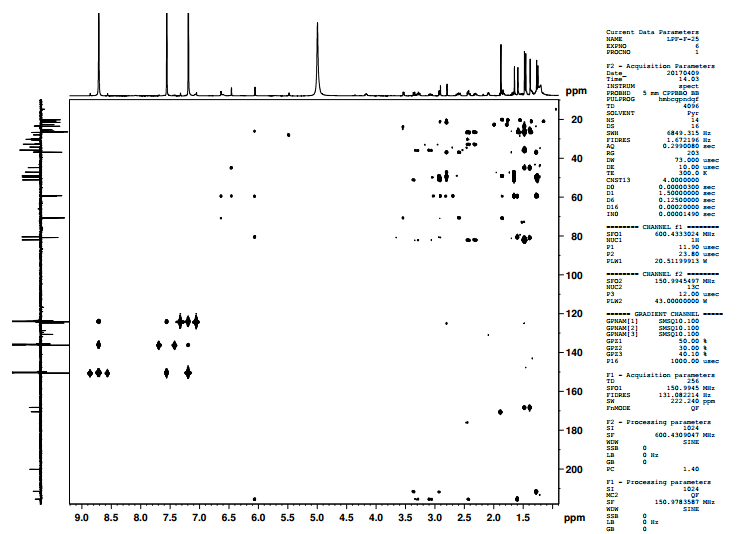


Figure S31. HMBC spectrum of the new compound **5**


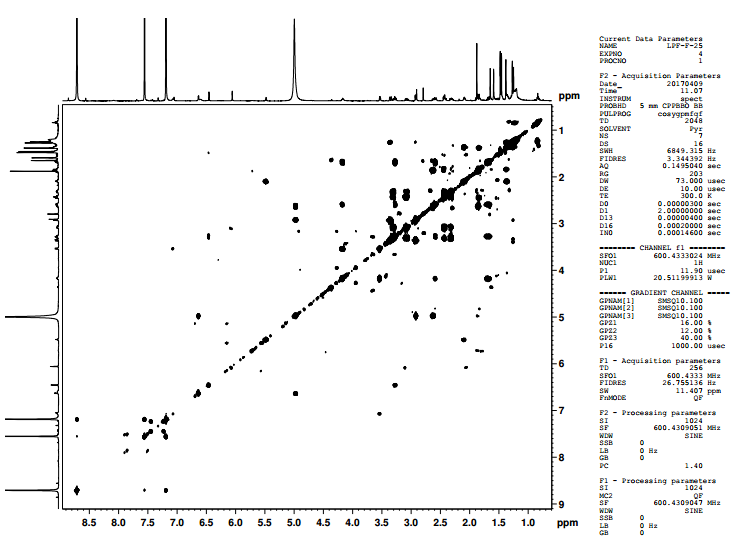


Figure S32. ^1^H-^1^H COSY spectrum of the new compound **5**

Figure S33. HR-ESI-MS spectrum of the new compound **5**.


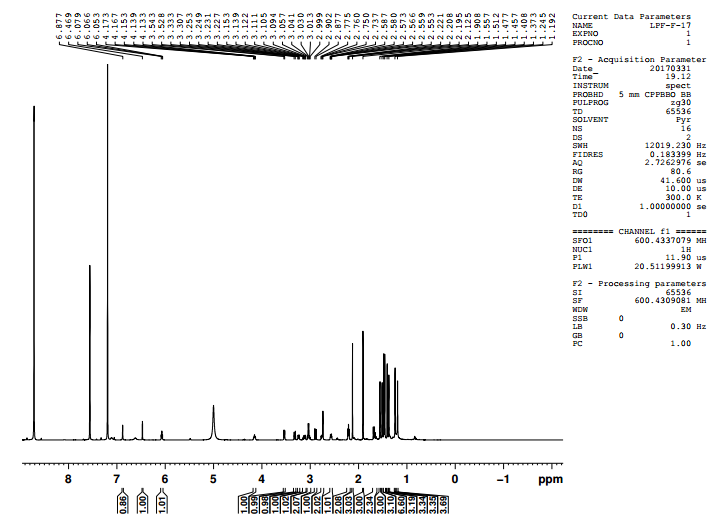


Figure S34. ^1^H-NMR (600 MHz, DMSO-*d*_6_) spectrum of the new compound **6**


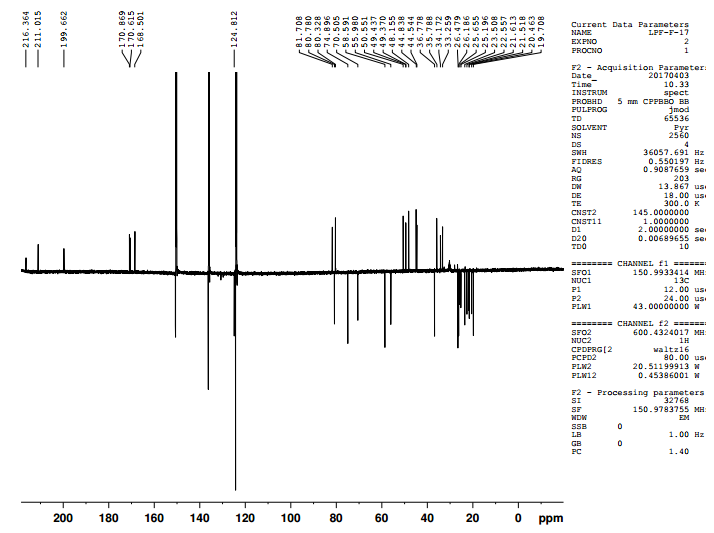


Figure S35. ^13^C-APT (150 MHz, DMSO-*d*_6_) spectrum of the new compound **6**


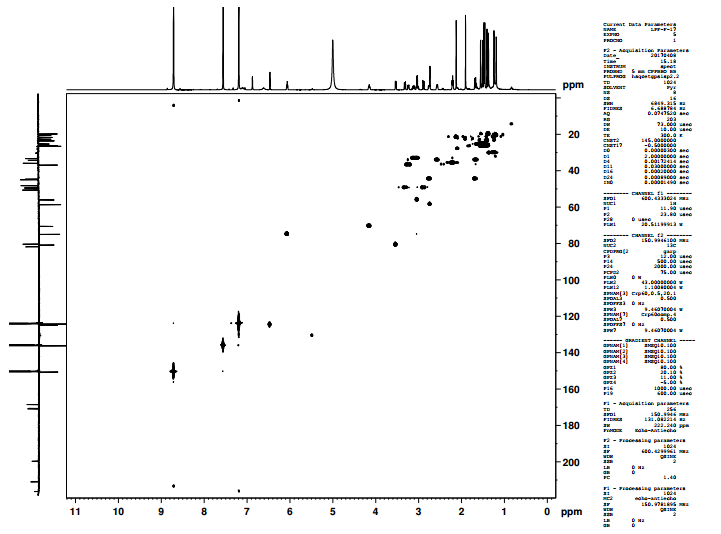


Figure S36. HSQC spectrum of the new compound **6**


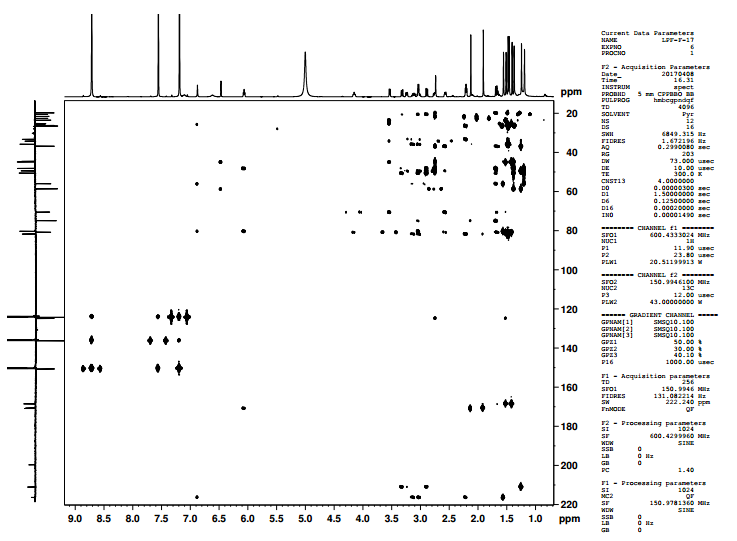


Figure S37. HMBC spectrum of the new compound **6**


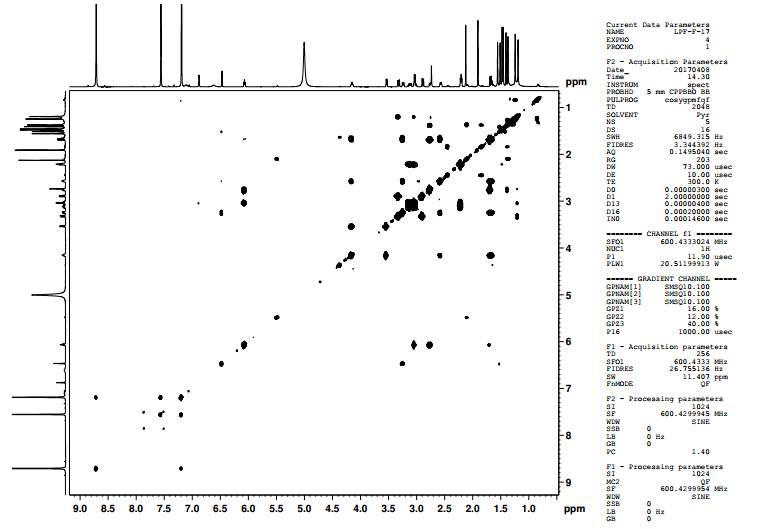


Figure S38. ^1^H-^1^H COSY spectrum of the new compound **6**

Figure S39. HR-ESI-MS spectrum of the new compound **6**.


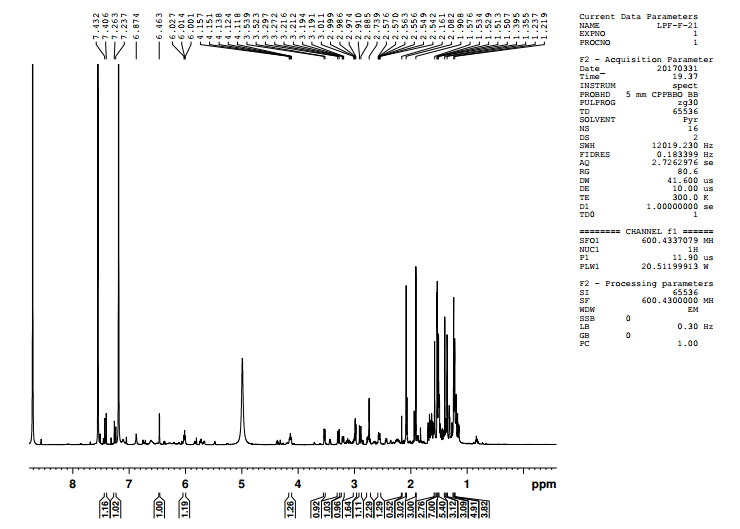


Figure S40. ^1^H-NMR (600 MHz, DMSO-*d*_6_) spectrum of the new compound **7**


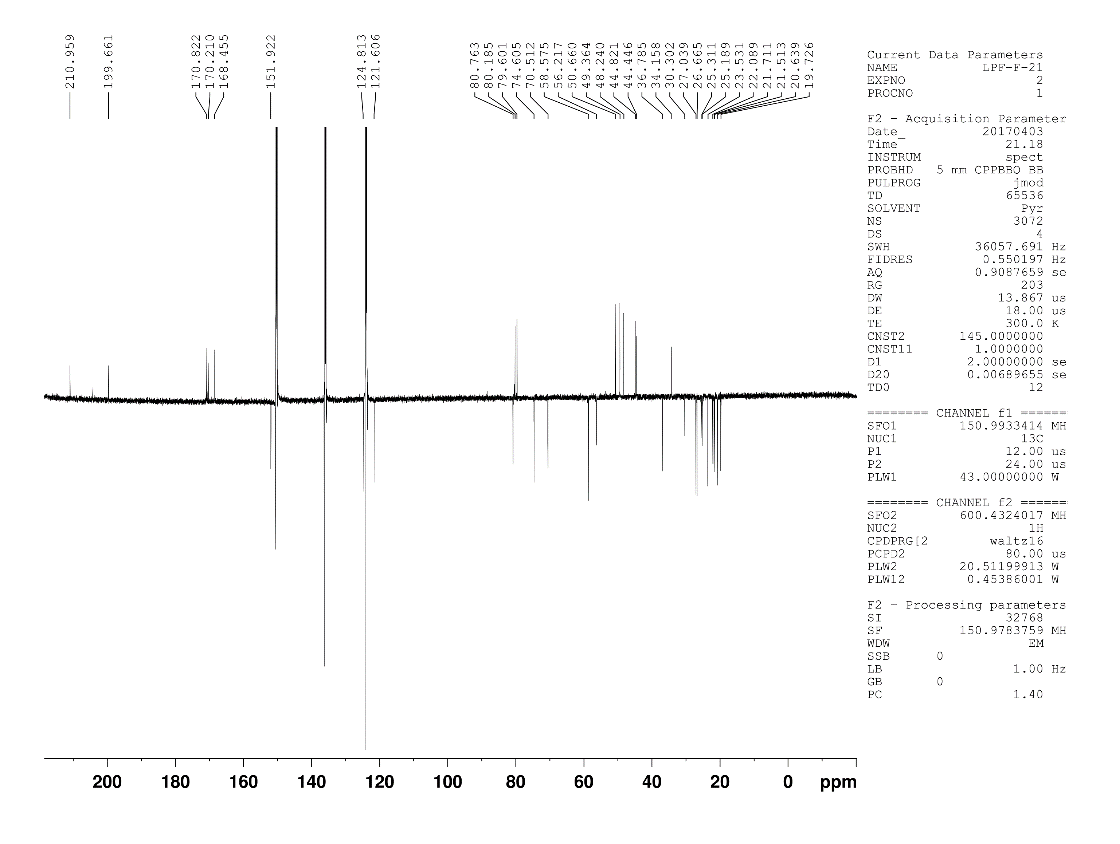


Figure S41. ^13^C-APT (150 MHz, DMSO-*d*_6_) spectrum of the new compound **7**


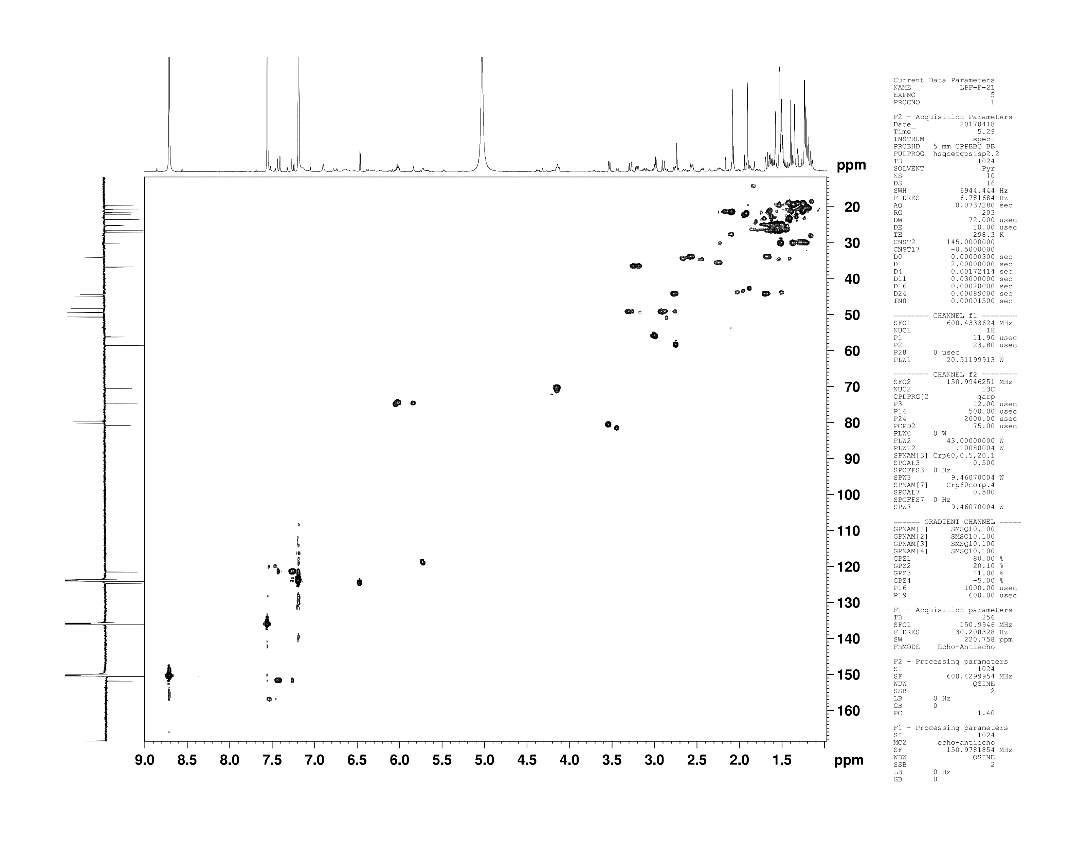


Figure S42. HSQC spectrum of the new compound **7**


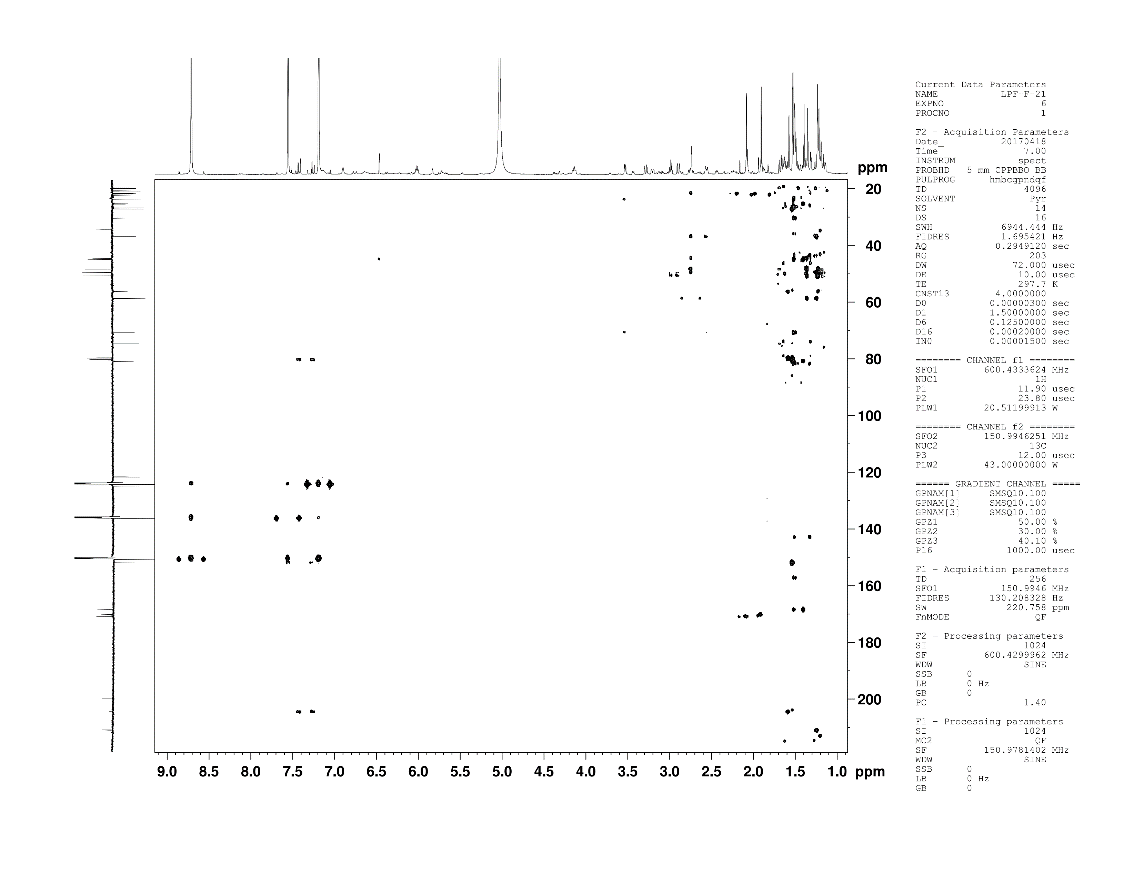


Figure S43. HMBC spectrum of the new compound **7**


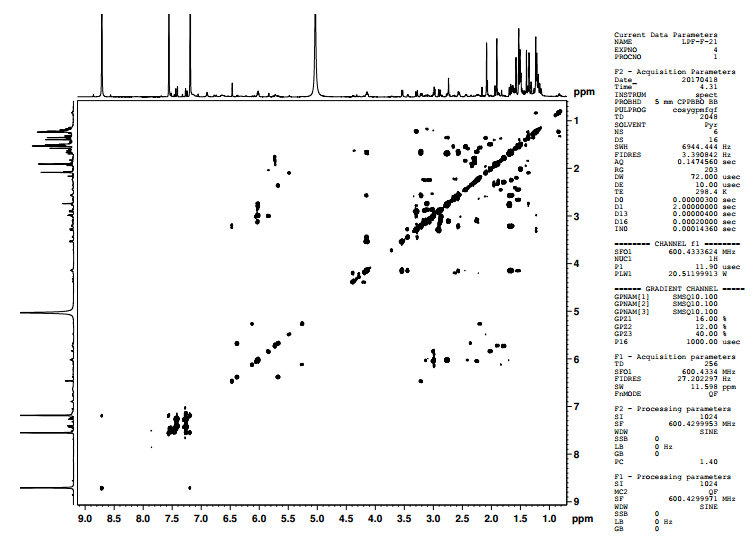


Figure S44. ^1^H-^1^H COSY spectrum of the new compound **7**

Figure S45. HR-ESI-MS spectrum of the new compound **7**.


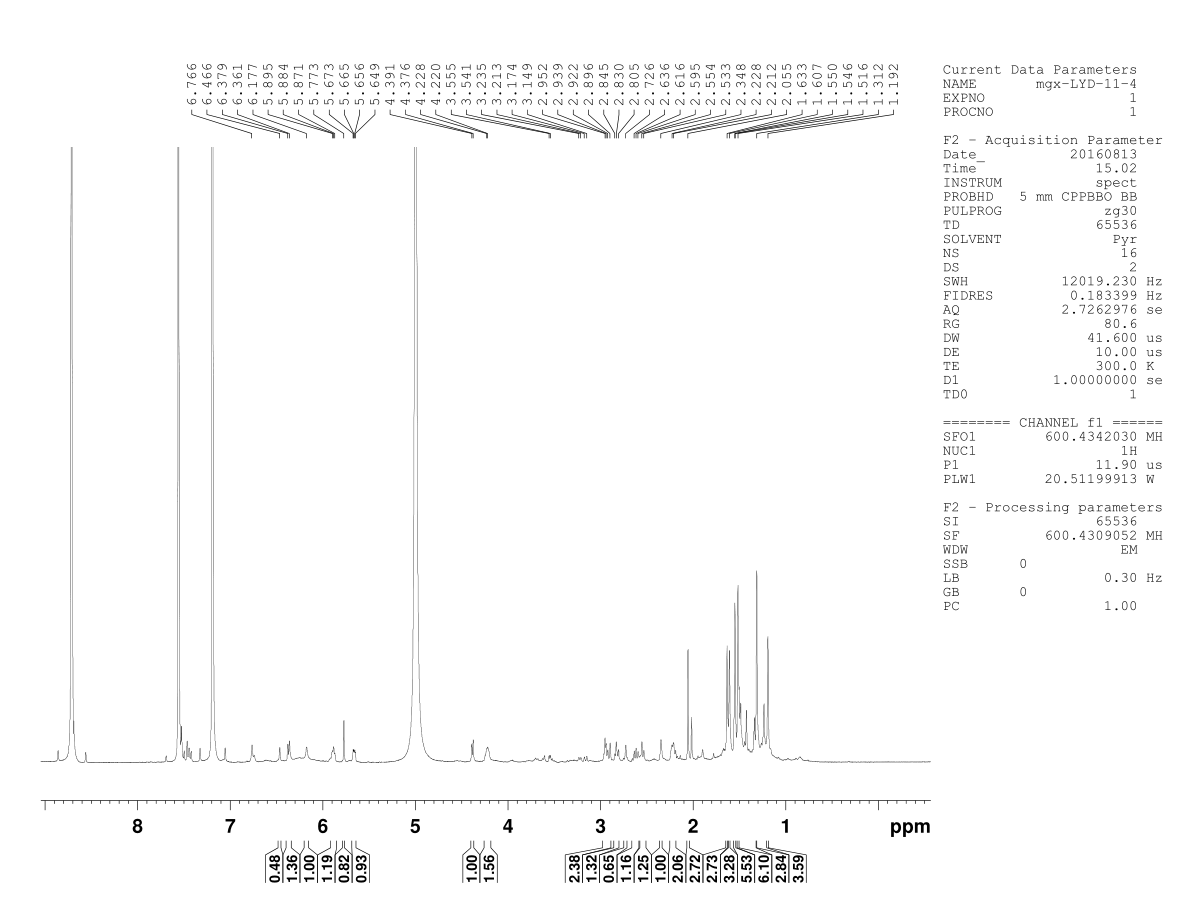


Figure S46. ^1^H-NMR (600 MHz, DMSO-*d*_6_) spectrum of the new compound **8**


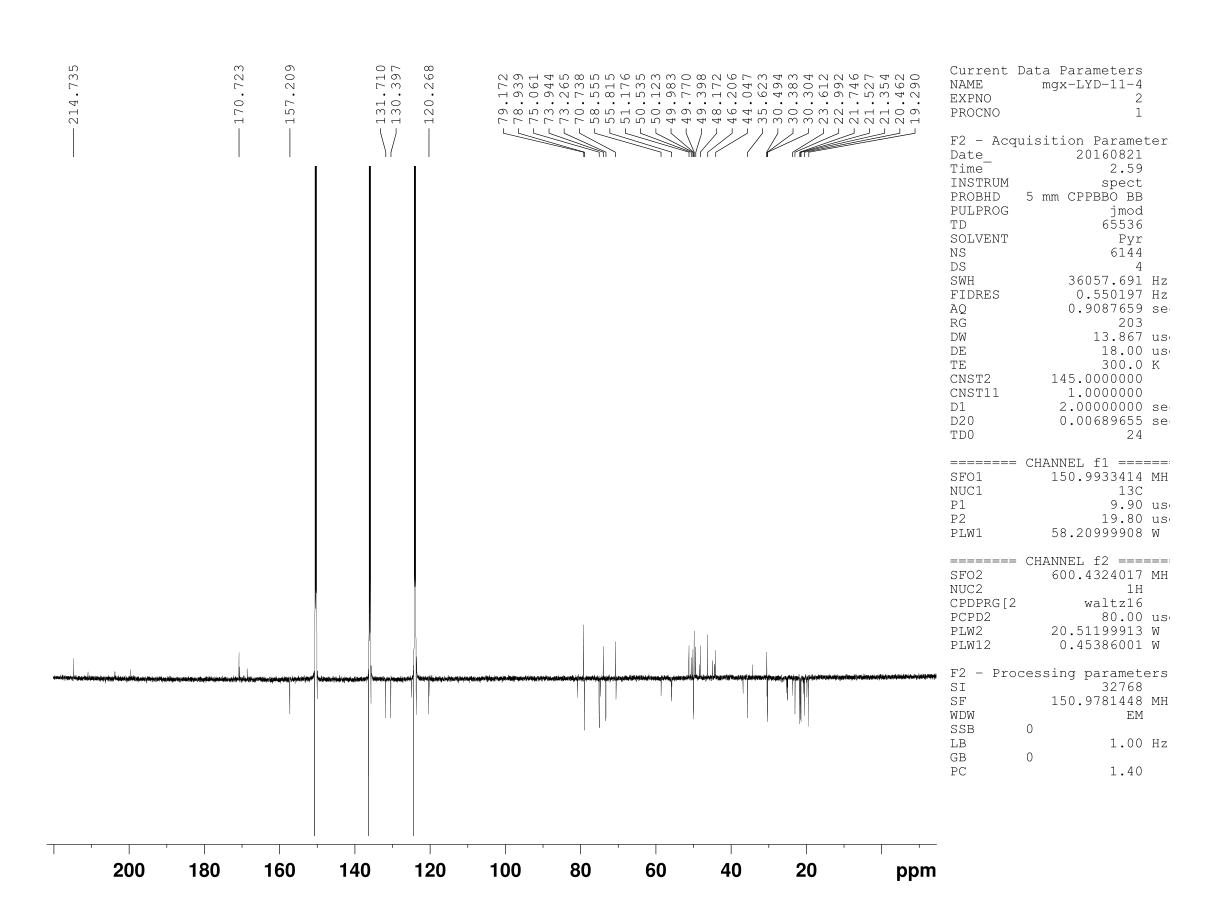


Figure S47. ^13^C-APT (150 MHz, DMSO-*d*_6_) spectrum of the new compound **8**


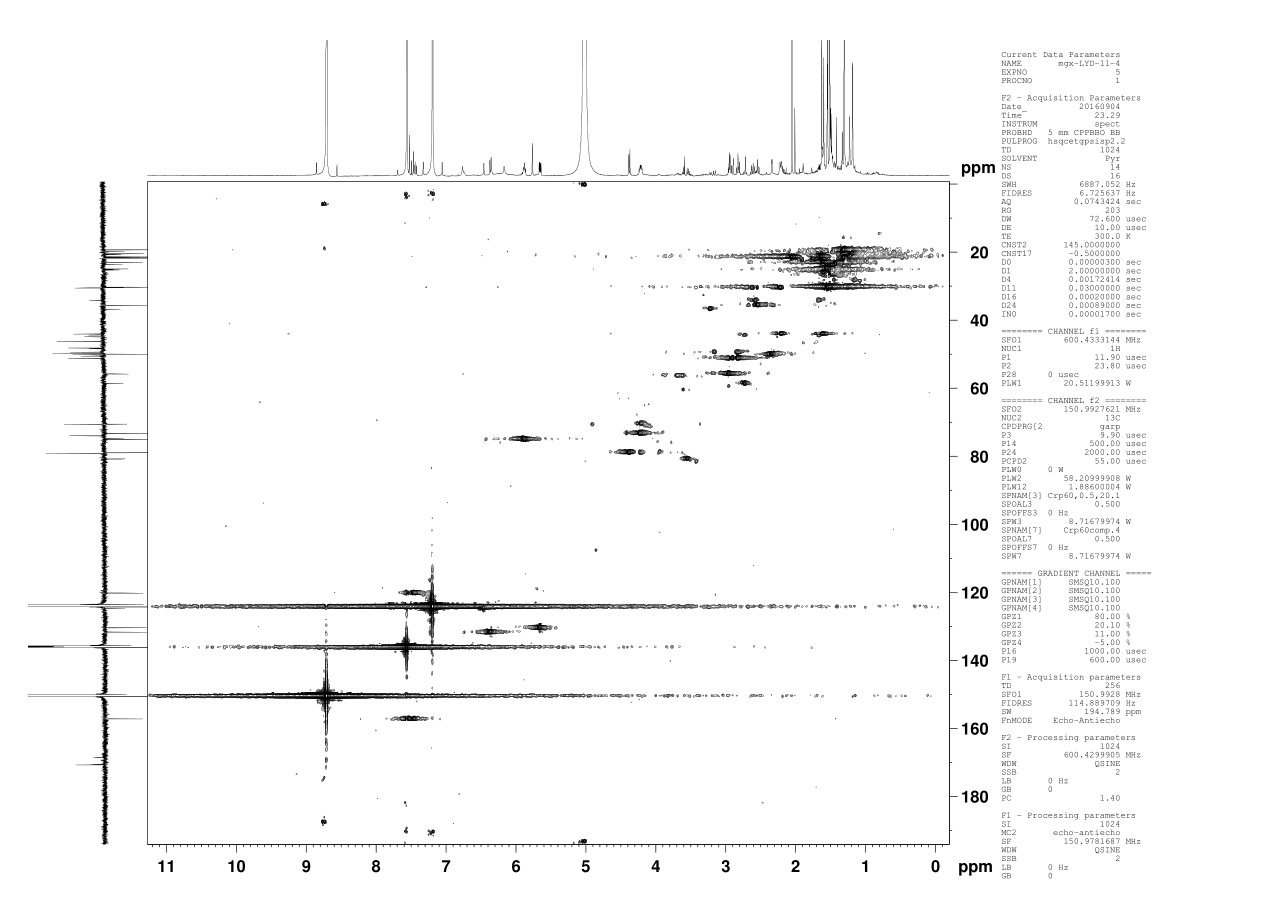


Figure S48. HSQC spectrum of the new compound **8**


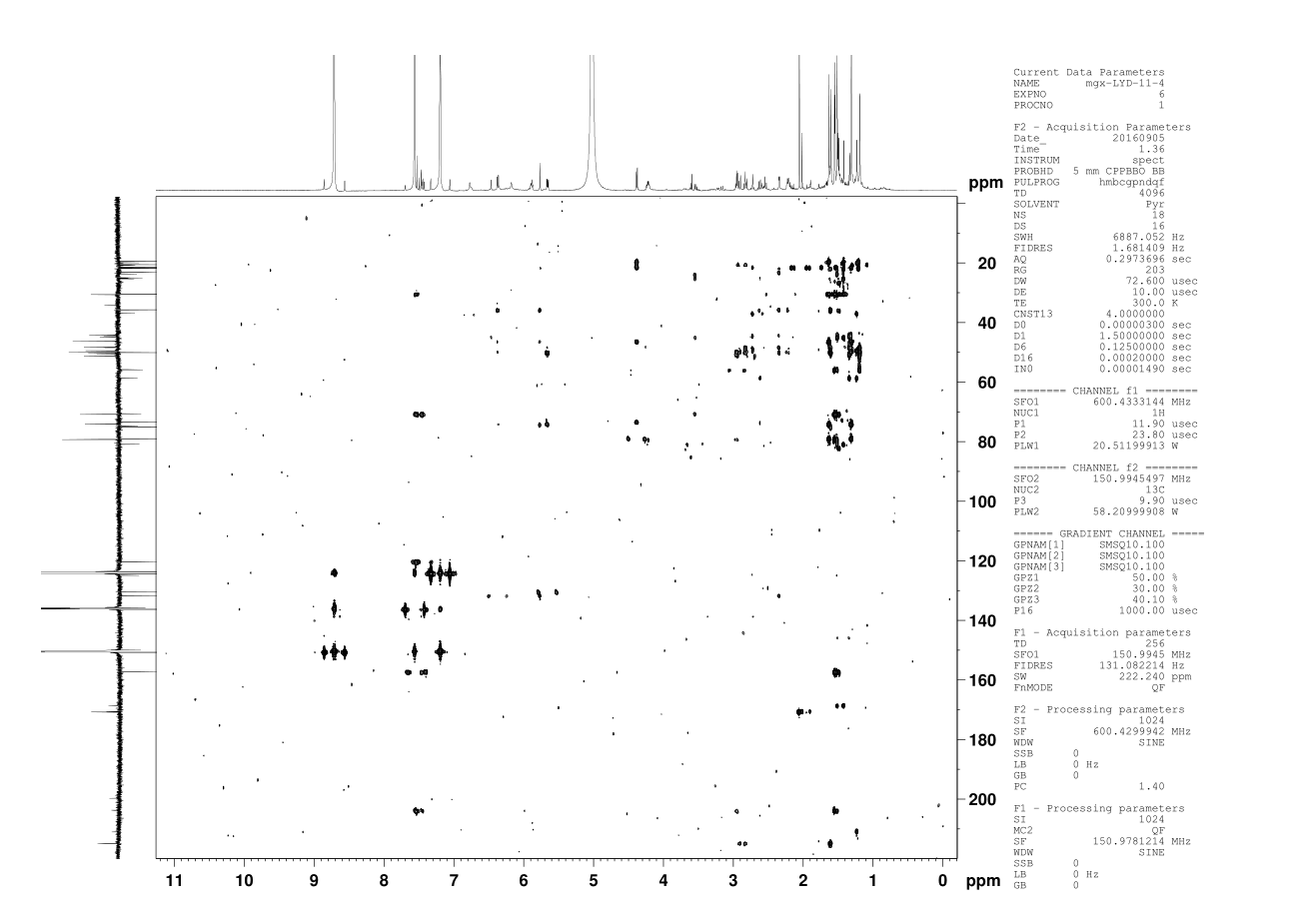


Figure S49. HMBC spectrum of the new compound **8**


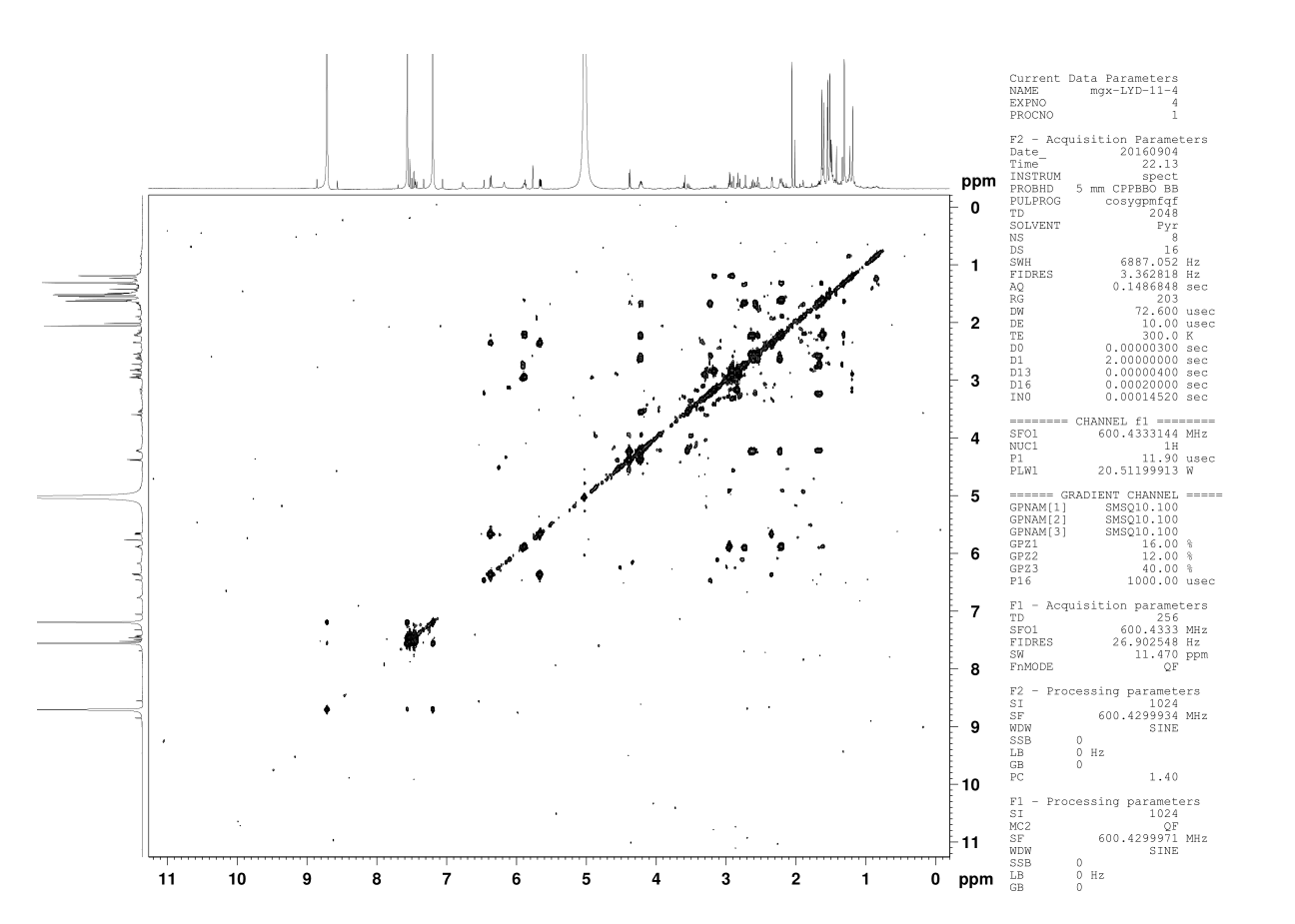


Figure S50. ^1^H-^1^H COSY spectrum of the new compound **8**

Figure S51. HR-ESI-MS spectrum of the new compound **8**.


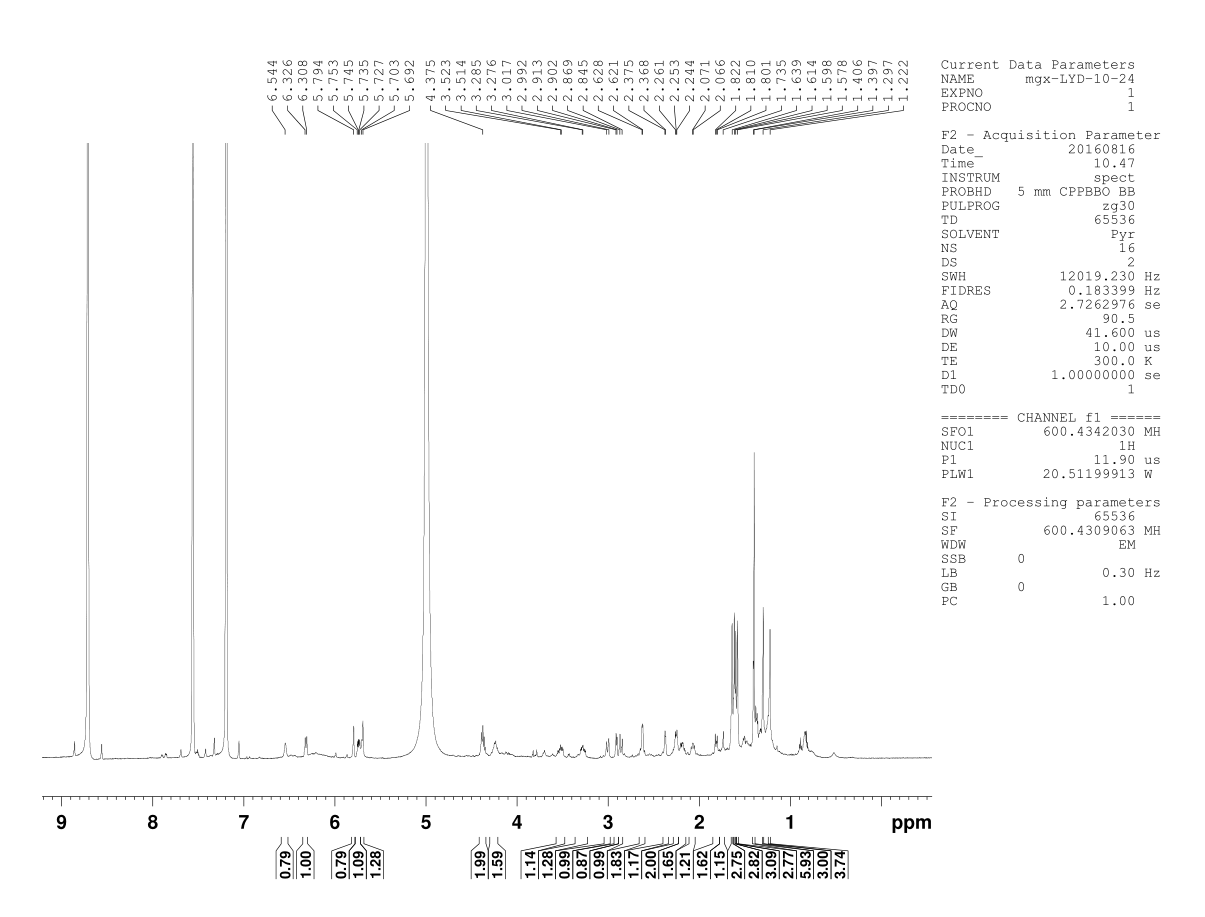


Figure S52. ^1^H-NMR (600 MHz, DMSO-*d*_6_) spectrum of the new compound **9**


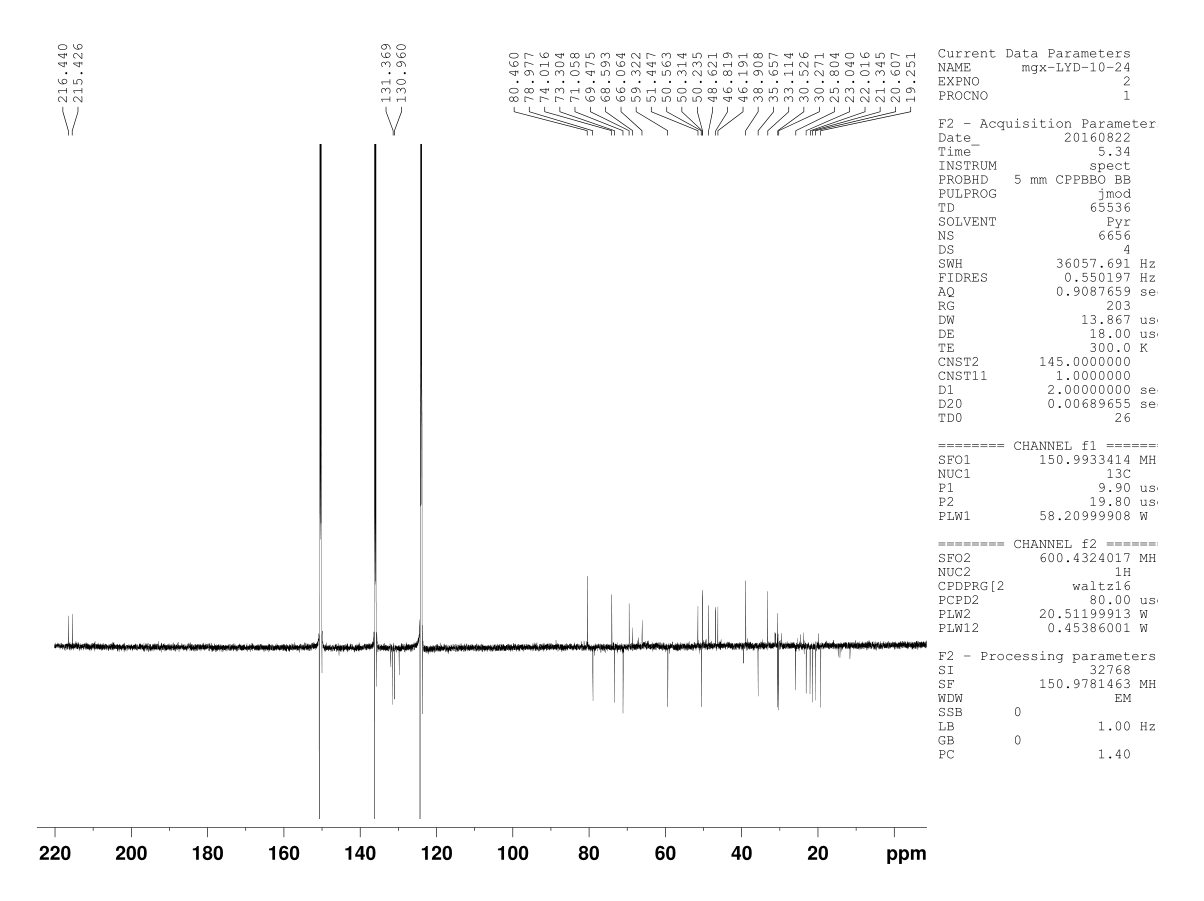


Figure S53. ^13^C-APT (150 MHz, DMSO-*d*_6_) spectrum of the new compound **9**


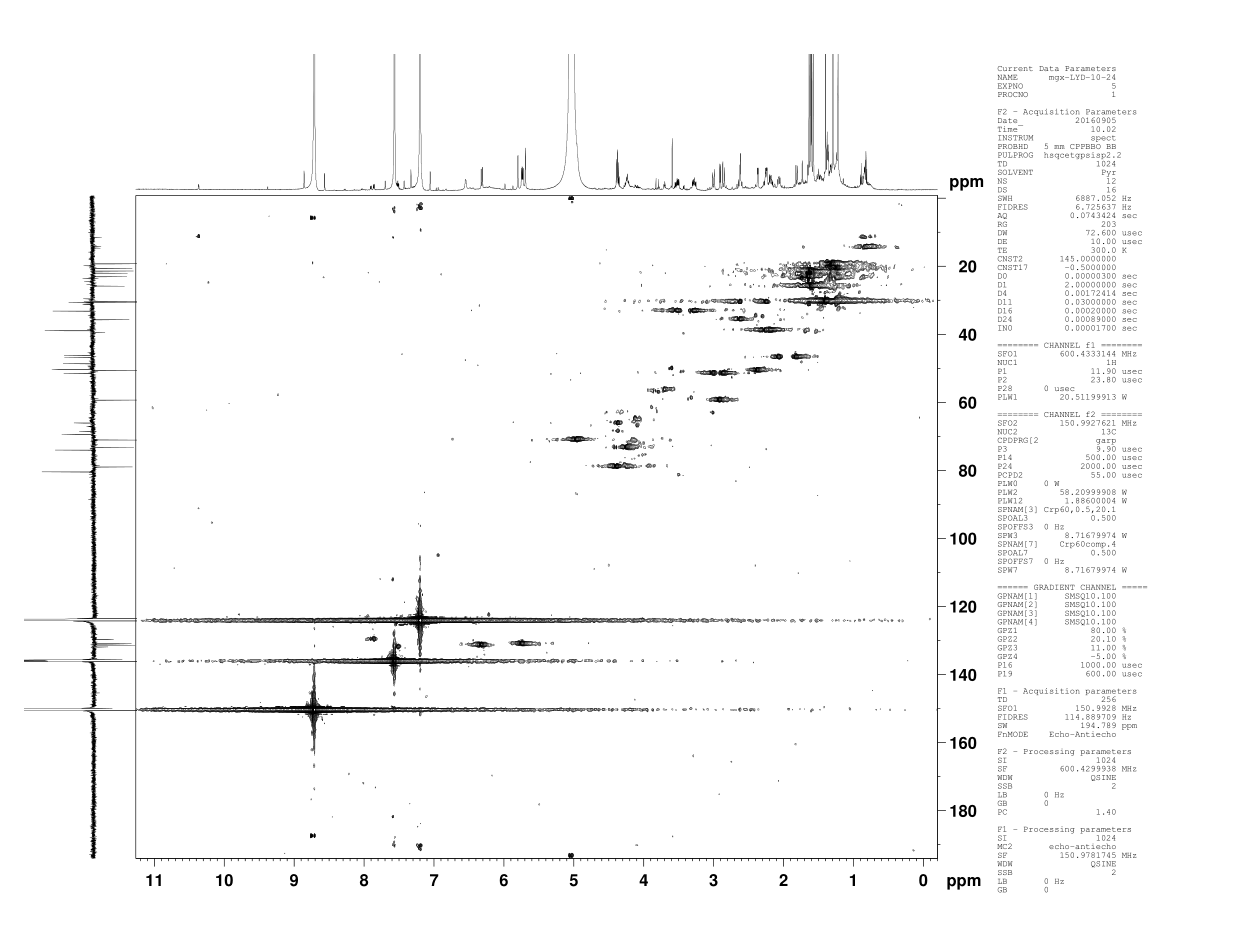


Figure S54. HSQC spectrum of the new compound **9**


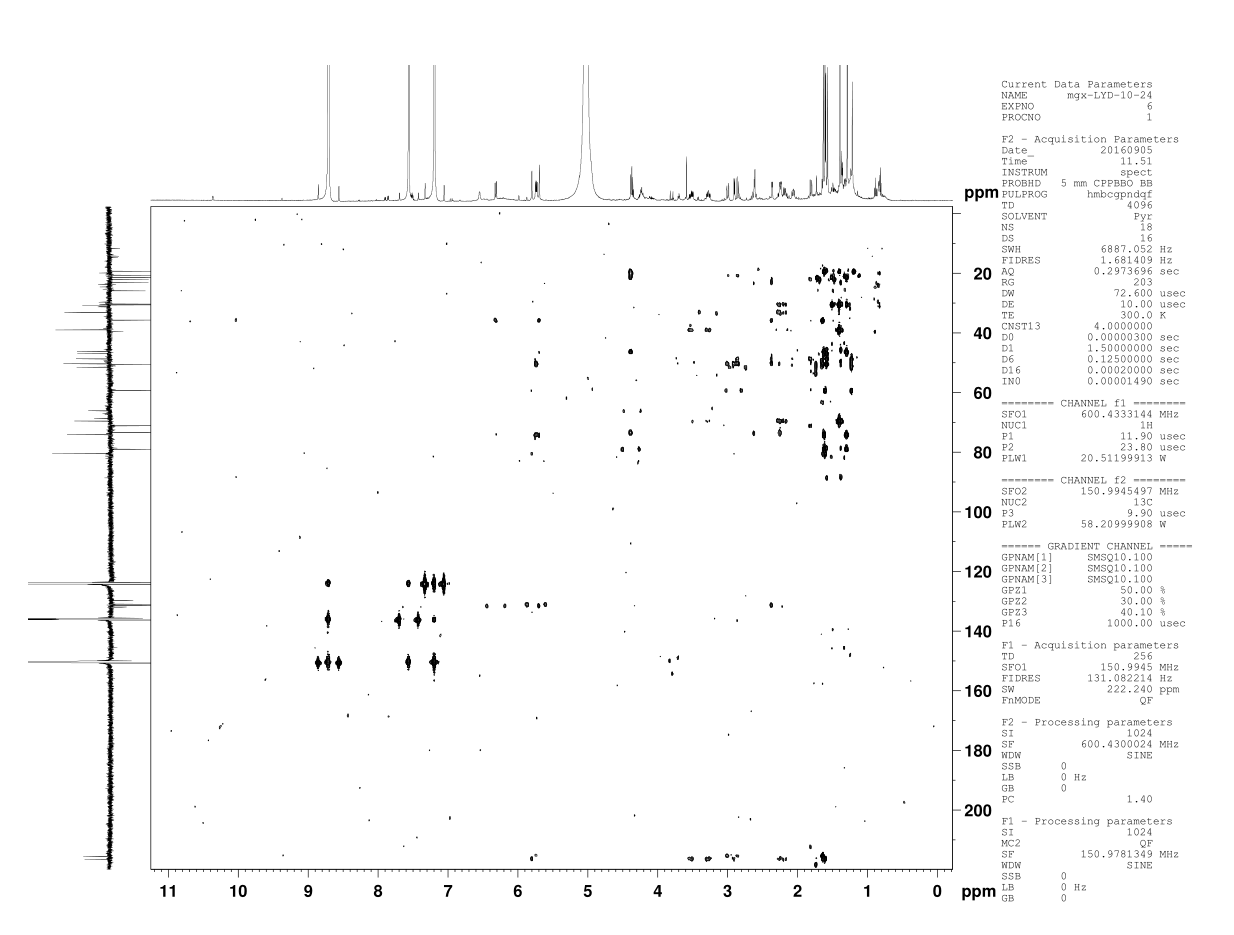


Figure S55. HMBC spectrum of the new compound **9**


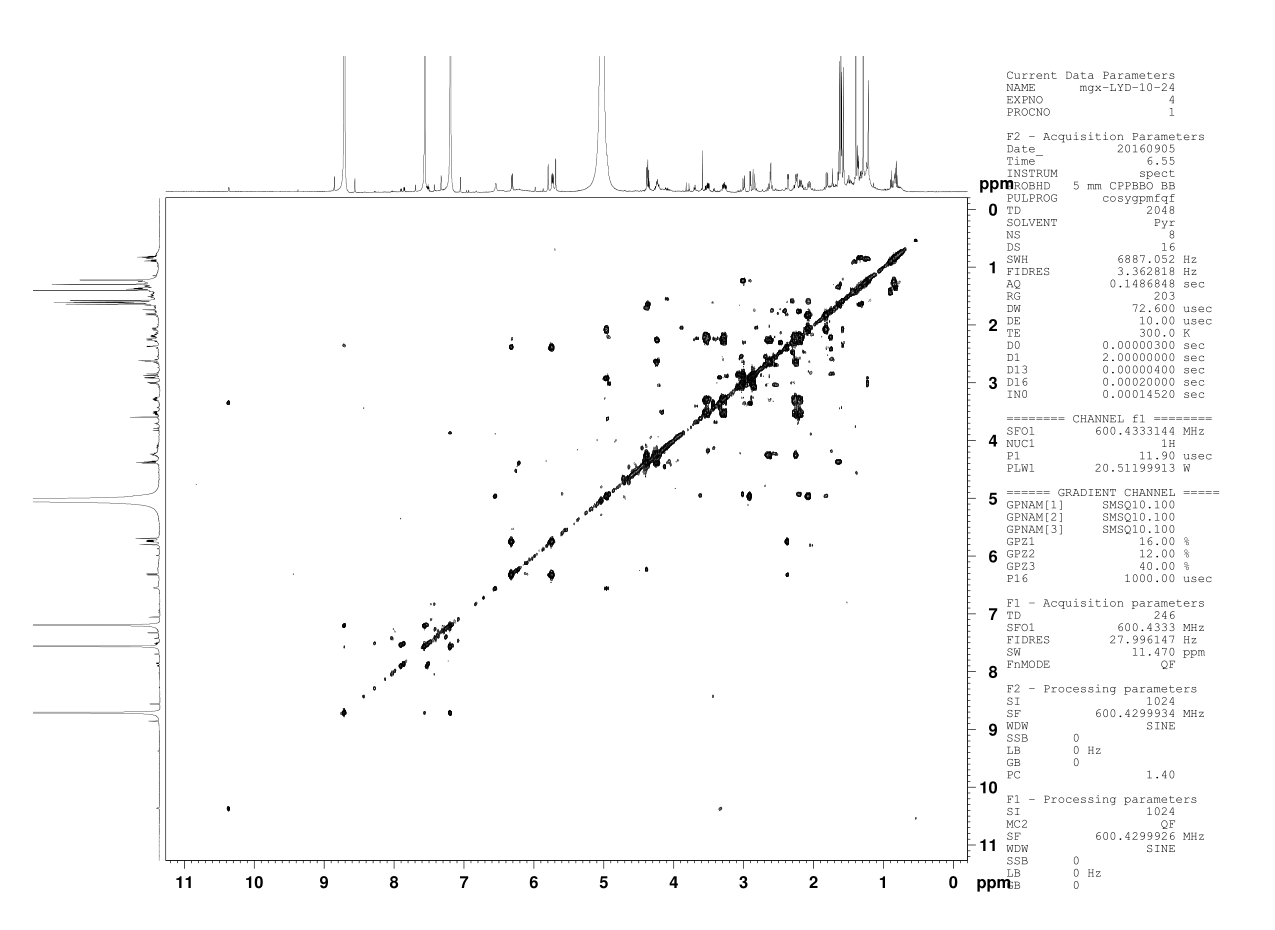


Figure S56. ^1^H-^1^H COSY spectrum of the new compound **9**

Figure S57. HR-ESI-MS spectrum of the new compound **9**.


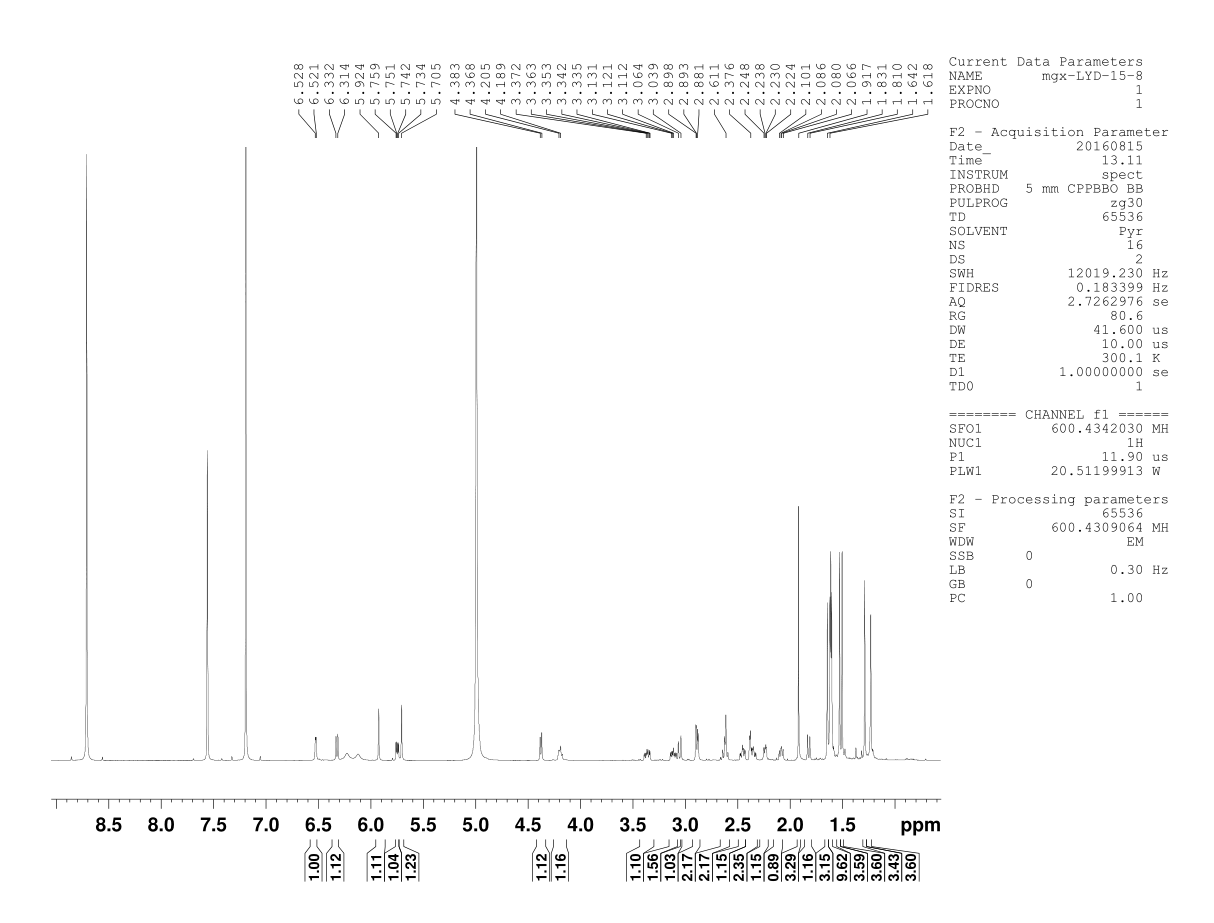


Figure S58. ^1^H-NMR (600 MHz, DMSO-*d*_6_) spectrum of the new compound **10**


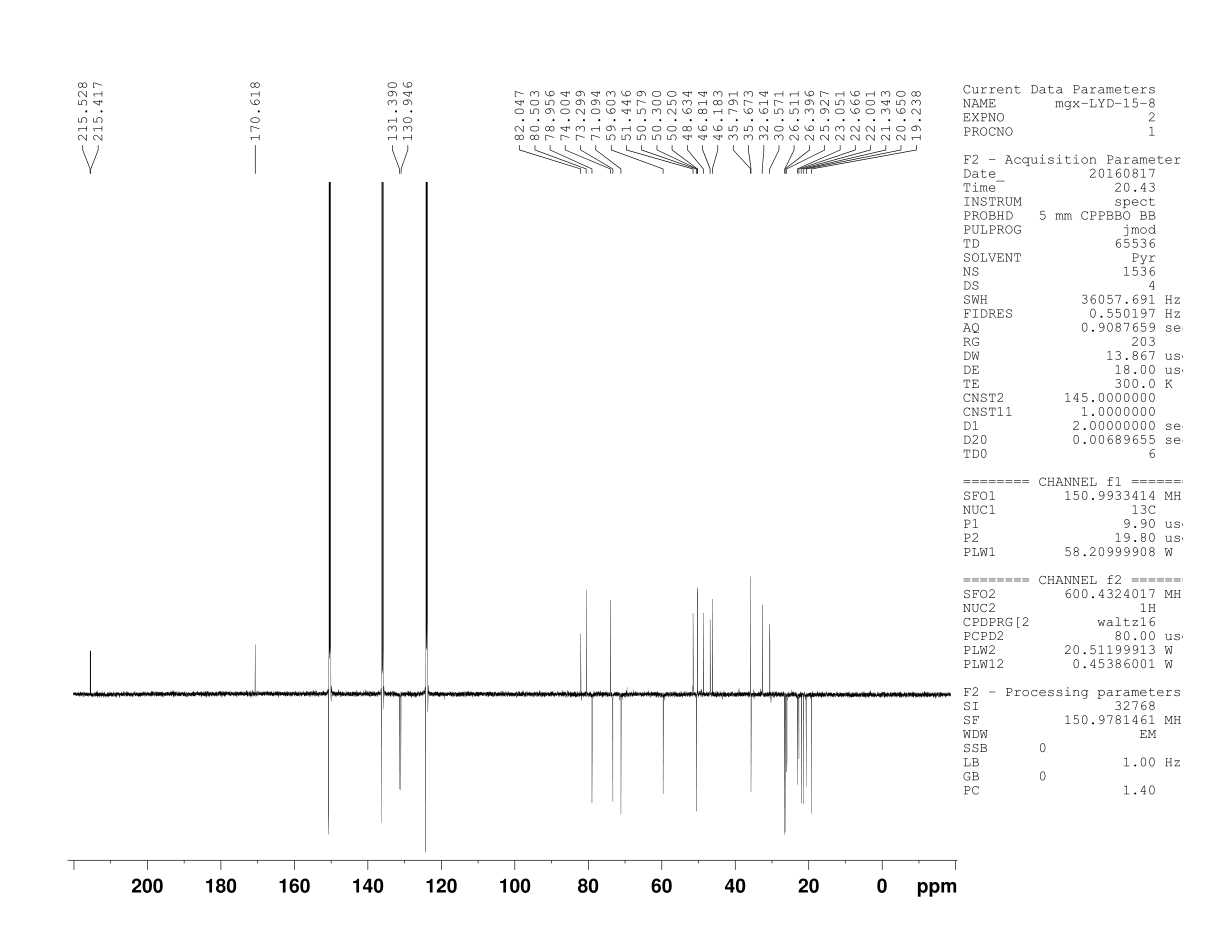


Figure S59. ^13^C-APT (150 MHz, DMSO-*d*_6_) spectrum of the new compound **10**


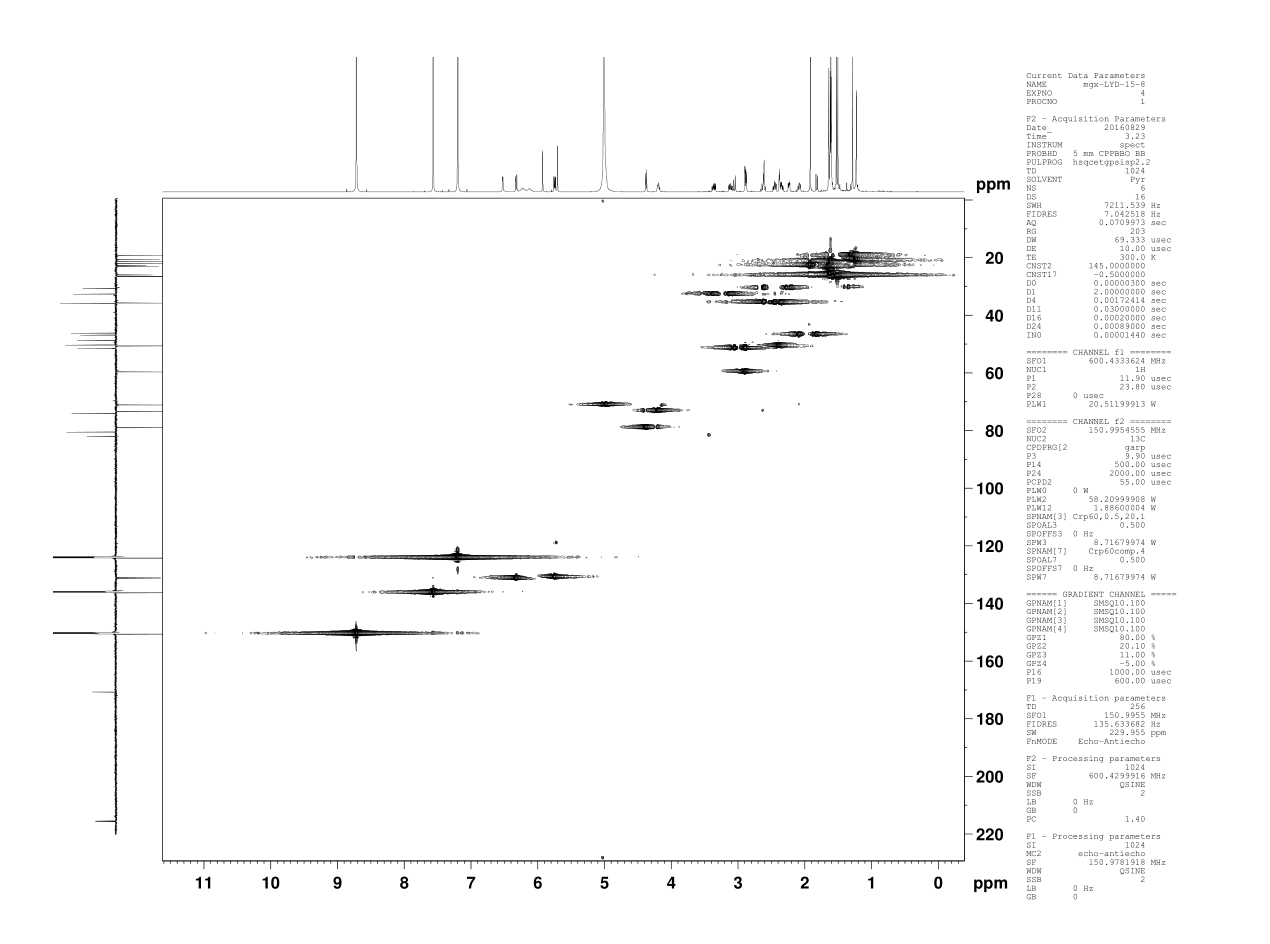


Figure S60. HSQC spectrum of the new compound **10**


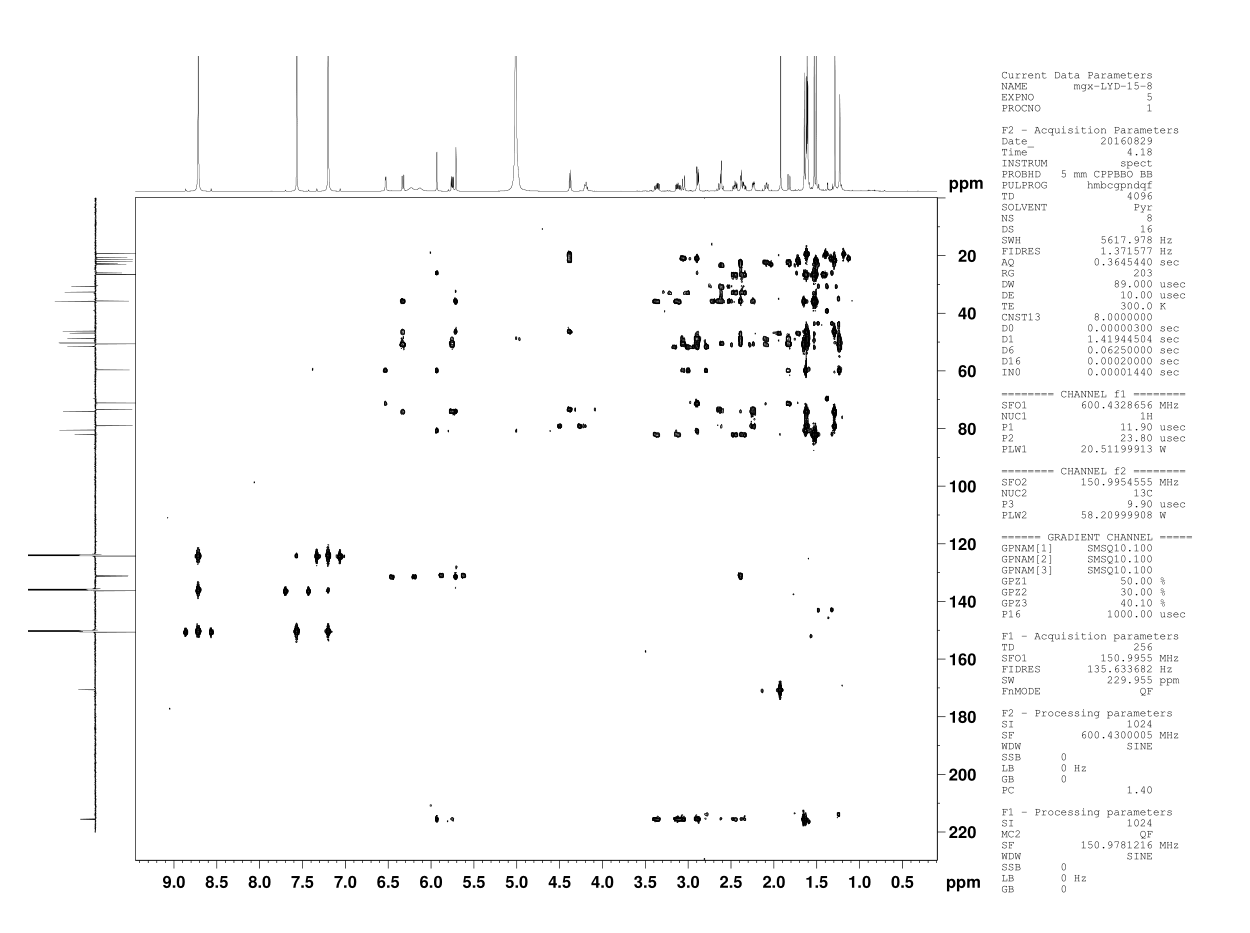


Figure S61. HMBC spectrum of the new compound **10**

Figure S62. HR-ESI-MS spectrum of the new compound **10**.

1. * Corresponding author. Tel./fax: +86 010 57833296.

   *E-mail address:* mgxfl98785@163.com (G.X. Ma); [xdxu2012@163.com](mailto:xdxu2012@163.com) (X. Xu)

   ^a^These authors contributed equally to this work. [↑](#footnote-ref-1)
